# Efficient Magneto-Luminescent Nanosystems based on Rhodamine-Loaded Magnetite Nanoparticles with Optimized Heating Power and Ideal Thermosensitive Fluorescence

**DOI:** 10.1021/acsami.2c14016

**Published:** 2022-10-27

**Authors:** Idoia Castellanos-Rubio, Ander Barón, Oier Luis-Lizarraga, Irati Rodrigo, Izaskun Gil de Muro, Iñaki Orue, Virginia Martínez-Martínez, Ainara Castellanos-Rubio, Fernando López-Arbeloa, Maite Insausti

**Affiliations:** † Departamento Química Orgánica e Inorgánica, Facultad de Ciencia y Tecnología, UPV/EHU, Barrio Sarriena s/n, 48940, Leioa, Spain; ‡ Departamento Electricidad y Electrónica, Facultad de Ciencia y Tecnología, UPV/EHU, Barrio Sarriena s/n, Leioa48940, Spain; § BC Materials, Basque Center for Materials, Applications and Nanostructures, Barrio Sarriena s/n, Leioa48940, Spain; ∥ SGIker, Servicios Generales de Investigación, UPV/EHU, Barrio Sarriena s/n, Leioa48940, Spain; ⊥ Departamento Química Física, Facultad de Ciencia y Tecnología, UPV/EHU, Barrio Sarriena s/n, Leioa48940, Spain; # Departamento Genética, Antropología Física y Fisiología Animal, Facultad de Medicina, UPV/EHU, Leioa48940, Spain; 7 Biocruces Bizkaia Health Research Institute, Cruces Plaza, Barakaldo48903, Spain; 8 Biomedical Research Center in Diabetes Network and Associated Metabolic Diseases, Madrid28029, Spain; 9 IKERBASQUE Basque Foundation for Science, Bilbao48013, Spain

**Keywords:** magnetite nanoparticles, shape control, magnetic hyperthermia, rhodamine, magneto-fluorescent, T-sensor

## Abstract

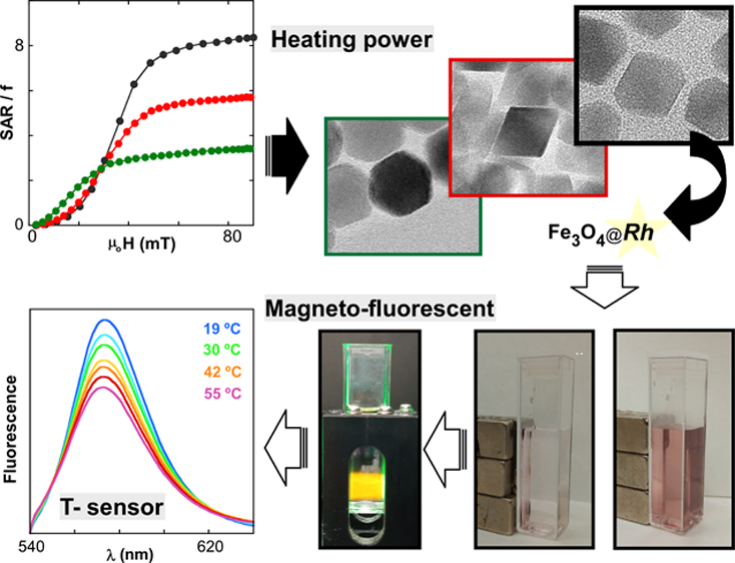

Nanosystems that simultaneously contain fluorescent and magnetic modules can offer decisive advantages in the development of new biomedical approaches. A biomaterial that enables multimodal imaging and contains highly efficient nanoheaters together with an intrinsic temperature sensor would become an archetypical theranostic agent. In this work, we have designed a magneto-luminescent system based on Fe_3_O_4_ NPs with large heating power and thermosensitive rhodamine (Rh) fluorophores that exhibits the ability to self-monitor the hyperthermia degree. Three samples composed of highly homogeneous Fe_3_O_4_ NPs of ∼25 nm and different morphologies (cuboctahedrons, octahedrons, and irregular truncated-octahedrons) have been finely synthesized. These NPs have been thoroughly studied in order to choose the most efficient inorganic core for magnetic hyperthermia under clinically safe radiofrequency. Surface functionalization of selected Fe_3_O_4_ NPs has been carried out using fluorescent copolymers composed of PMAO, PEG and Rh. Copolymers with distinct PEG tail lengths (5–20 kDa) and different Rh percentages (5, 10, and 25%) have been synthesized, finding out that the copolymer with 20 kDa PEG and 10% Rh provides the best coating for an efficient fluorescence with minimal aggregation effects. The optimized Fe_3_O_4_@Rh system offers very suitable fluorescence thermosensitivity in the therapeutic hyperthermia range. Additionally, this sample presents good biocompatibility and displays an excellent heating capacity within the clinical safety limits of the AC field (≈ 1000 W/g at 142 kHz and 44 mT), which has been confirmed by both calorimetry and AC magnetometry. Thus, the current work opens up promising avenues toward next-generation medical technologies.

## 1 Introduction

The innovative functionalization of magnetic nanoparticles (MNPs) keeps offering groundbreaking paths within the theranostic field.^1−3^ Nanoprobes that contain dual imaging modalities are able to provide more reliable diagnosis and reduced data processing times.^4,5^ Specifically, nanosystems with the capacity to combine magnetic resonance imaging (MRI) (well-known for the deep tissue penetration/good spatial resolution) with luminescence imaging (widely used due to its high sensitivity/temporal resolution) can yield multiplexed images safely.^6,7^ Furthermore, MNP-based systems can be targeted into specific tissues or cells through noninvasive magnetic targeting (MT),^8^ which can lead to specific drug-delivery^9^ or to tumor-selective therapy such as magnetic hyperthermia (MH).^10^

So far, the preferred magnetic phase for nanobiotechnological applications such as MT and MH is the mixed-valence magnetite (Fe_3_O_4_) because of its good magnetic performance, low toxicity, and good biodegradability.^11,12^ The heating power of Fe_3_O_4_ NPs greatly depends on the morphology, size, monodispersity, and aggregation degree of the NPs. Previous studies have proven that the magnetothermal efficiency of magnetite NPs can be maximized by tuning the shape anisotropy of NPs above the superparamagnetic limit (>20 nm) while minimizing the dipolar interactions.^13,14^ On the other hand, PEGylation technology, using high molecular weight molecules (≥5 kDa), provides good colloidal stability in physiological conditions and prevents the formation of MNP clusters.^15^ Avoiding agglomeration effects is also of utmost importance when using organic luminophores because the self-aggregation-mediated quenching can largely restrict the luminophores’ practical application.^16,17^ In addition, the close proximity between luminophores and iron oxide cores leads to a strong emission quenching due to the large UV–vis absorption and scattering of Fe_3_O_4_ NPs, and these aggregation effects become more critical as the size of the MNPs increases.^5^ Since organic dyes are more sensitive to aggregation and scattering effects, they have started to be replaced by quantum dots and lanthanide complexes in the fabrication of fluorescent nanocomposites,^18,19^ but at the expense of major toxicity. Therefore, the incorporation of magnetic and optical moieties into a common nanosystem that holds out high biocompatibility, optimal hyperthermia performance, and efficient photoluminescence remains a challenge. Decisive progress would be attained with the integration of a biocompatible fluorophore whose emissive properties do not depend on the pH or ionic strength but do change with the temperature.^20,21^ This is the case for rhodamine 3B, whose fluorescence emission is insensitive to a saline environment and pH-independent above 6 and, in addition, presents a fluorescence quantum yield that linearly decreases with temperature.^22,23^ The thermosensitivity of rhodamine 3B makes it a proper local molecular thermometer, which could result in an adventurous module in a theranostic system composed of nanoheaters.

We present herein a promising magneto-optic platform with high heating power, potential for image-multiplexing, and capacity for self-monitoring the temperature increase. First, we have analyzed the magnetic properties of three types of Fe_3_O_4_ NPs of around 25 nm and different morphologies in order to choose the magnetic core that is able to attain the best magnetothermal performance. Second, we coated the optimal magnetic core with a copolymer composed of poly(maleic anhydride-*alt*-1-octadecene) (PMAO), 5-TAMRA cadaverine (an amine-modified rhodamine 3B with a hydrocarbon spacer of 5 C atoms), and poly(ethylene glycol)-amine (PEG-NH_2_). The suitability of the polymeric surface (molecular weight of the PEG and the amount of rhodamine grafted) has been studied to select the system with the most suitable optical properties. Finally, the thermal dependence of the fluorescence emission and the heating capacity of the best nanoplatform has been determined both in distillate water and physiological media.

## 2 Results and Discussion

### 2.1 Choice of the Most Suitable Magnetic Core

**Figure 1:**
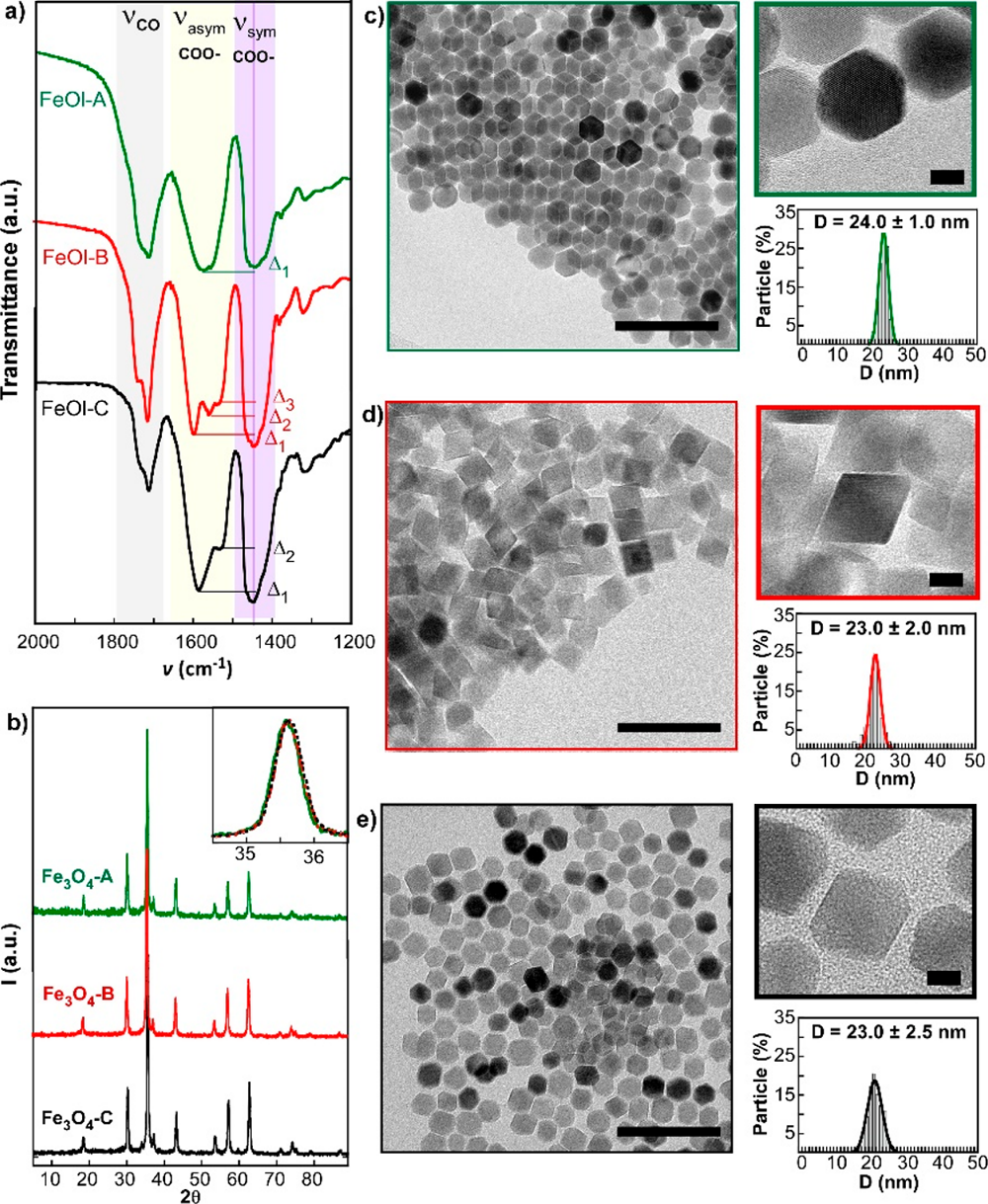
(a) FTIR spectra of iron oleate complexes (FeOl-A, FeOl-B, and FeOl-C). Stretching modes of the carbonyl group ν(C=O) are within gray region, asymmetric and symmetric ν(COO^–^) bands are within yellow and purple regions, respectively, and Δ = ν_asym_ – ν_sym_ have been marked with straight lines. The FTIR spectra in the 4000–400 cm^–1^ range are in Figure S1. (b) X-ray powder diffraction patterns of Fe_3_O_4_ NPs samples (A, B, and C). (c–e) TEM micrographs and corresponding size distributions of samples A, B, and C, respectively. Large scale bars: 100 nm. Zoom scale bars: 10 nm.

Theranostic platforms based on the magnetothermal effect of MNPs should be composed of reliable heat nanoinductors. It is of utmost importance that the MNPs present high purity, crystallinity, and size/shape homogeneity so that the heat generation is systematic and reproducible. Thermal decomposition of iron-oleate is one of the best considered wet-chemical synthesis method to produce iron oxide NPs with sizes over 20 nm.^24^ This method has recently been systematically refined to obtain single nanocrystals of Fe_3_O_4_ with bulk-like magnetic properties and minimal size/shape polydispersities.^25^ It is widely known that the shape of the MNPs has a key role in the hyperthermia performance^26^ and recent works have suggested an increased magnetic anisotropy in NPs with octahedral morphology when compared to cuboctahedral NPs.^13,27^ In order to further investigate the impact of NP shape on their magnetic properties, three Fe_3_O_4_ samples (A, B, and C) with the same average dimension (23–24 nm) and different morphology (cuboctahedrons, octahedrons, and irregular truncated-octahedrons) have been synthesized using three different iron oleates (FeOl). As shown in Table 1, the FeOl precursors were annealed at different conditions (temperature and time) (see Materials and Methods), which causes an evident change in the metal-carboxylate coordination types, and consequently, in the morphology of the magnetite NPs. Figure 1a displays the FTIR spectra of FeOl-A, FeOl-B, and FeOl-C in the range 2000–1200 cm^–1^ range. As described in previous works, the distance between the asymmetric and symmetric ν(COO^–^) bands (Δ = ν_asym_ – ν_sym_) provides information about the nature of the metal-carboxylate coordination.^28^ The precursor that was annealed for longer (FeOl-A: 60 h at 110 °C) presents a single separation between ν(COO^–^) bands (Δ_1_ = 130 cm^–1^), indicating a bridging type coordination. On the contrary, FeOl-B and FeOl-C precursors (annealed during 21 h) present a combination of bridging (110 cm^–1^ < Δ < 200 cm^–1^) and bidentate (Δ < 110 cm^–1^) coordinations (see Table 1). Finally, the precursor that was annealed at a higher temperature (FeOl-C: 120 °C) presents a clear reduction of the carbonyl (C=O) band (stretching modes at 1711 and 1736 cm^–1^ marked in gray in Figure 1a), which is related to noncoordinated or weakly coordinated ligands.^29,30^

**Table 1:** Summary of Synthesis Conditions and Samples Features^a^

sample	annealing *T* of FeOl (°C)	annealing *t* of FeOl (hours)	*d* between ν(COO^–^)Δ_1_, Δ_2_, Δ_3_ (cm^–1^)	molar ratio O.A:FeOl	XRD_NPs_*D* (σ) (nm)	TEM_NPs_*D* (σ) (nm)
**A**	110	60	130	2.2:1	25 (1)	24.0 (1.0)
**B**	110	21	150, 110, 80	2:1	25 (1)	23.0 (2.0)
**C**	120	21	140, 85	2:1	25 (2)	23.0 (2.5)

a The annealing temperature (*T*) and time (*t*) of the FeOl precursor prior to NP synthesis, the distance (*d*) between the asymmetric and symmetric ν(COO^–^) bands (Δ = ν_asym_ – ν_sym_) of the FTIR spectra of FeOl complexes, the molar ratio of oleic acid and iron oleate (O.A: FeOl) in the Fe_3_O_4_ NP synthesis, the crystallite size (*D*_XRD_) calculated by the Scherrer equation, and the particle mean dimension obtained by TEM (*D*_TEM_).

In order to synthesize magnetite NPs of comparable average size, we decomposed FeOl precursors following the same heating profile (see Materials and Methods). Powder X-ray diffraction (XRD) of the NPs (samples A, B, and C) showed the inverse spinel structure of magnetite (PDF #880866) with no trace of wüstite or other phases (see Figure 1b). The calculated crystalline sizes of the three samples (see Table 1 and Table S1) are around 25 nm and completely match with the sizes determined by TEM analysis, which means that the three samples are composed of single nanocrystals of the same average dimension.

The TEM micrographs and the corresponding histograms of samples A, B, and C are presented in Figure 1c–e, respectively, where two clear features can be observed: (i) very narrow size distributions and (ii) distinct morphologies. Sample A is composed of extremely regular cuboctahedrons as a result of decomposing FeOl-A that presents just one coordination mode (bridging) (see Figure 1a). This single mode of medium strength tends to hinder any preferential growth. Additionally, when a molar ratio oleic acid:FeOl > 2 is used in the synthesis (Table 1), the growth rate of {111} and {100} facets become nearly equal, favoring cuboctahedron-like morphology. On the contrary, when the iron oleate precursor displays more than two coordination modes (which is the case of FeOl-B) and the molar ratio oleic acid:FeOl employed in the synthesis is ≤2, the nanoparticles are prone to present a well-defined octahedral shape (sample B) because the more reactive {100} planes tend to grow to extinction.^13^ On the other hand, the FeOl-C precursor (with a double splitting in the asymmetric ν(COO^–^) band, Figure 1a) gives rises to an intermediate morphology between cuboctahedron and octahedron. As can be observed in the micrographs of Figure 1e, sample C is composed of irregularly truncated octahedral NPs. This irregular truncation could be related to the low amount of weakly coordinated ligand in the FeOl-C.

**Figure 2:**
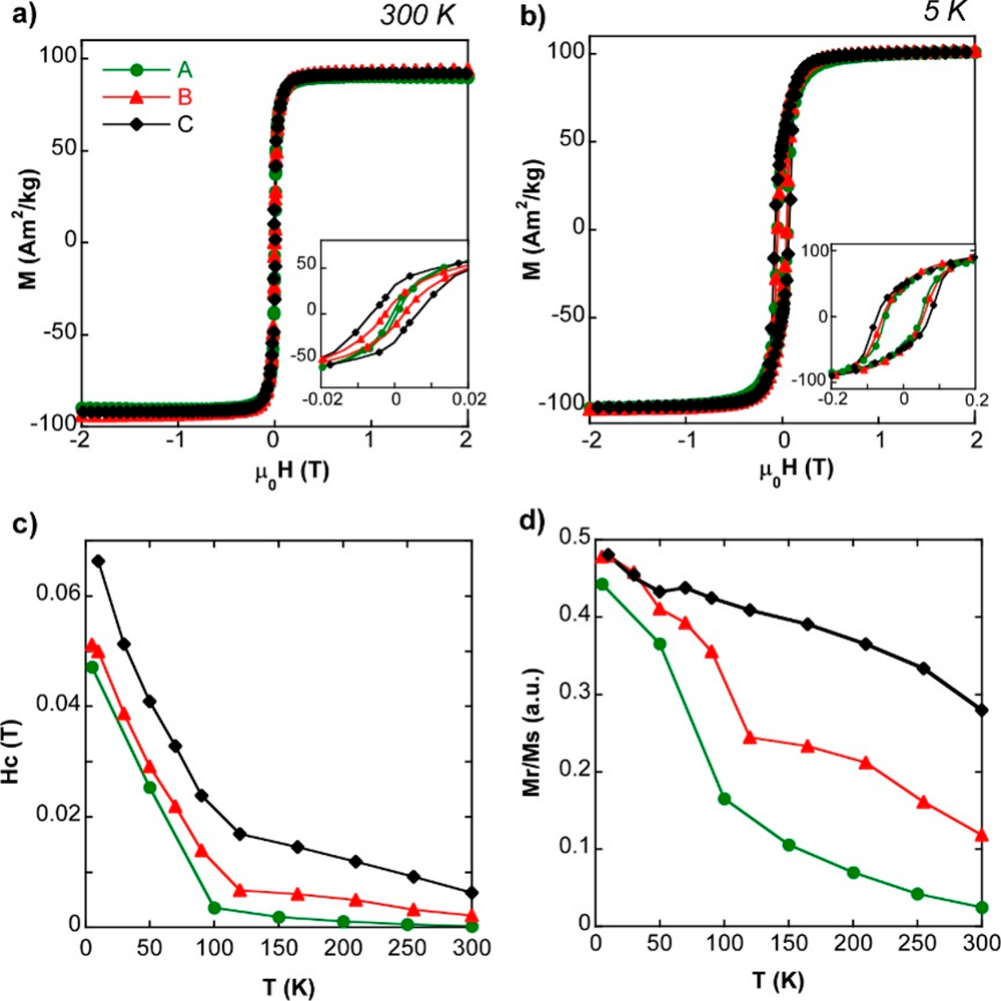
M (H) curves of samples A, B and C at **a)** 300 K and **b)** 5 K. **c)** Hc (T) evolution and **d)** Mr/Ms (T) evolution of samples A (green), B (red) and C (black).

The basic magnetic characterization performed by standard DC magnetometry pinpoints those magnetic properties that are strongly influenced by the morphology of the magnetic cores. At this point, we have particularly focused on the saturation magnetization (*M*_s_), coercive field (*H*_c_), and reduced remanent magnetization (*M*_r_/*M*_s_). The corresponding numerical data and *M*(*H*) curves at room temperature (300 K) and at low temperature (5 K) are presented in Table 2 and Figure 2a, b, respectively, while the whole thermal evolution is plotted in Figure 2c . Samples A, B, and C share a relevant common feature: saturation magnetization and the overall shape of the hysteresis loops do not change significantly across samples (considering the uncertainty of the measurement) (see Figure 2a, b). This is important because it confirms that nanoparticles have very similar chemical composition; that is, nearly stoichiometric magnetite. This conclusion is also supported by the fingerprint left by the Verwey transition on the ZFC/FC measurements (Figure S3), an easily detectable magnetization step that happens in the 100–110 K range in the three samples. Additionally, hysteresis loops at 5 K presented in Figure 2b (where the magnetic anisotropy is quite large and thermal fluctuations do not exist) resemble that of the magnetic single domains, so they can be fairly simulated within the Stoner-Wolhfarth model of uniaxial single domains (Figure S4). However, the impact of the morphology becomes quite significant when looking toward the coercive field and/or the remanence. Sample C presents much higher coercive field and remanence than samples A and B at any temperature; in fact, the gap between them keeps roughly constant with temperature. Given that the shape magnetic anisotropy largely contributes to the effective magnetic anisotropy, this result agrees with previous TEM analysis in which sample C has been described as being composed of irregular shaped particles with the highest aspect ratio (Figure 1e). This being so, hysteresis area is expected to be enhanced at any temperature because the shape anisotropy contribution is roughly temperature-independent. In the same vein, the differences in hysteresis observed between samples A and B can also be attributed to differences in morphology: cuboctahedral particles of sample A are more spherical than octahedral particles of sample B, so the latter are expected to have a larger effective magnetic anisotropy.^27^

**Table 2:** Summary of Saturation Magnetization (*M*_s_), Coercivity (*H*_c_), and Reduced Remanence (*M*_r_/*M*_s_) of Samples A, B, and C Obtained from the Hysteresis Loops at 300 and 5 K^a^

sample	*M*_s_ at RT (A·m^2^/kg_Fe3O4_)	*M*_s_ at 5 K (A·m^2^/kg_Fe3O4_)	H_c_ (mT) at RT	*H*_c_ (mT) 5 K	*M*_r_/*M*_s_ at RT	*M*_r_/*M*_s_5 K
A	90 (2)	101 (2)	0.2 (1)	47.1 (5)	0.03 (1)	0.45 (1)
B	94 (2)	101 (2)	2.1 (1)	51.2 (5)	0.12 (1)	0.48 (1)
C	92 (2)	101 (2)	6.3 (1)	66.3 (5)	0.28 (1)	0.48 (1)

a The *M*_s_ at RT and 5 K are normalized per unit mass of magnetite (Figure S2).

Therefore, the significant hysteresis enhancement in sample C should produce higher specific absorption rate (SAR) in magnetic hyperthermia. AC magnetometry experiments have corroborated that this is the case, as it will be demonstrated in the following.

**Figure 3:**
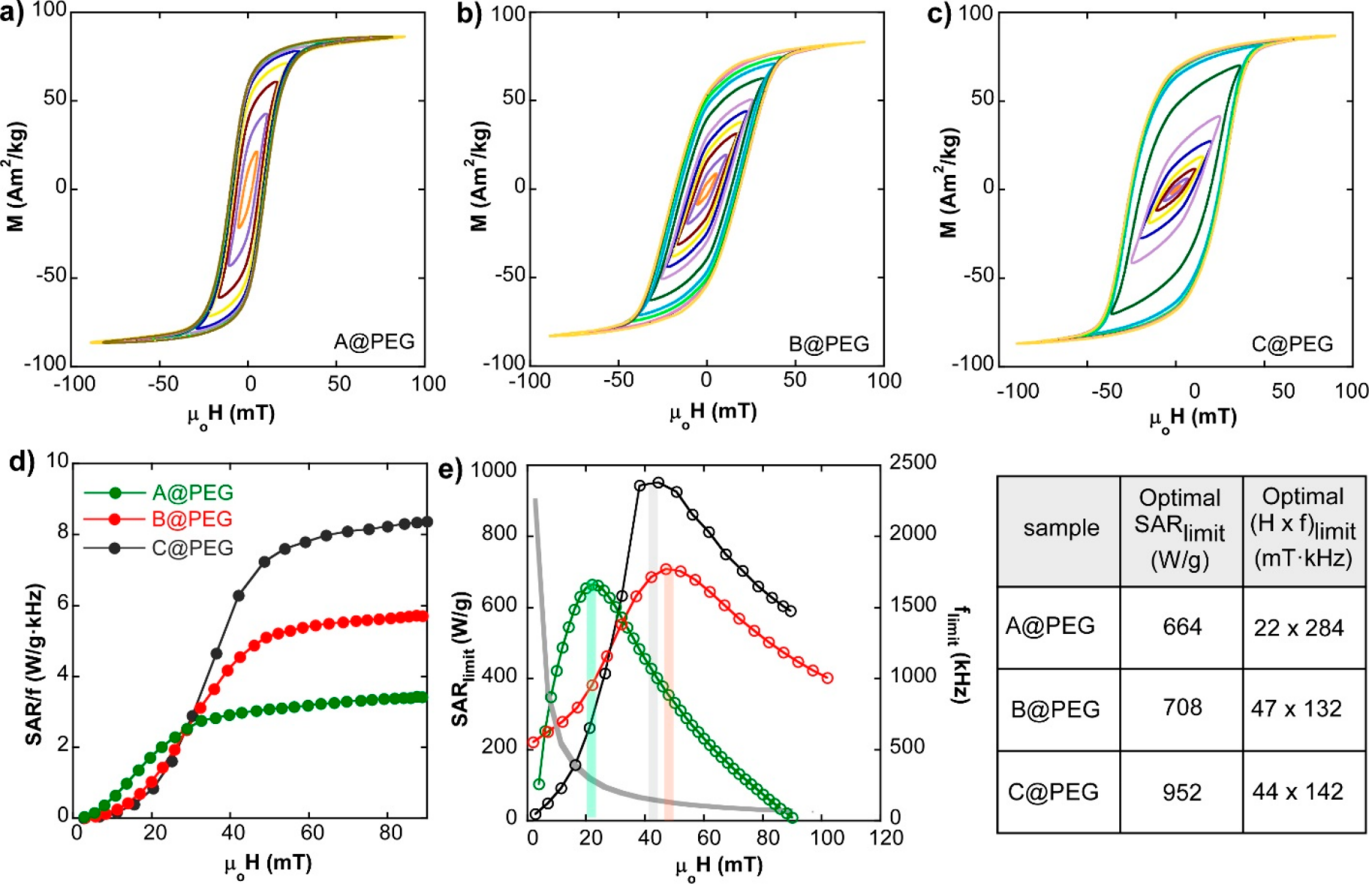
AC hysteresis loops in D.I. H_2_O and at 133 kHz of samples: (a) A@PEG, (b) B@PEG, and (c) C@PEG. (d) Experimental SAR/*f* versus field curves (at 133 kHz) for samples A@PEG, B@PEG, and C@PEG (the corresponding curves at 304 and 634 kHz are displayed in Figure S5). (e) Maximum achievable SAR and SAR_limit_ under the Hergt criterion for A@PEG, B@PEG, and C@PEG samples. The gray curve is the acceptable maximum frequency, *f*_limit_(*H*) = 5 × 10^9^/*H*, for a given magnetic field intensity. The intersection of the gray curve and the colored bar of each sample shows the optimal frequency for obtaining the maximum SAR_limit_ (values are collected in the adjacent table).

For magnetic hyperthermia applications, it is essential to carry out an appropriate surface modification of the NPs to make them stable in aqueous media. A functional way to enhance the colloidal stability of the NPs without impairing the magnetic properties is to conduct a proper surface coating with PMAO-PEG copolymer composed of long PEG tails.^15^Figure 3a–c show AC hysteresis loops of PEGylated A, B, and C samples at 133 kHz. Dynamical hysteresis loops with increasing magnetic field amplitudes show that the hysteresis area enhances markedly from sample A to sample C, when the applied magnetic excitation is above ∼30 mT. According to single magnetic domain models, the SAR and/or the hysteresis area should saturate at larger values of the excitation field as the effective magnetic anisotropy increases. A previous work demonstrated that the excellent response of magnetite nano-octahedra as heat producers is related to the shape magnetic anisotropy characteristic of this shape.^13^ As stated before, slightly different degrees of truncation/irregularities in the octahedral-like shape of the nanoparticles (which is the case of sample C) should lead to larger AC hysteresis area. This effect can also be noticeable when the magnetic cores have a larger tendency to form chains.

As long as there are not substantial dipolar interactions among NPs, the AC hysteresis area versus field curve (SAR(*H*)) approaches a step-like function. So the SAR(*H*) curve is quite small below certain threshold field amplitude, which is close to the corresponding coercive field. As a consequence, SAR/*f*(*H*) curves of samples A@PEG, B@PEG, and C@PEG, displayed in Figure 3d, show that the maximum SAR (or saturation SAR) of sample C@PEG experiences a remarkable increase relative to samples A@PEG and B@PEG. It is well-known that for biomedical applications the magnetic excitation must be kept under safe conditions, such as that imposed by the Hergt criterion,^31^ which establishes the limiting condition of *Hf* = 5 × 10^9^ A·m^–1^·s^–1^. Thus, considering the Hergt threshold, the SAR_limit_(H) curves have been obtained following a recently published approach.^32^ But in this case, the curves of SAR versus AC magnetic field amplitude, corresponding to different excitation frequencies, have been fitted to a single sigmoidal-type function (see Model S1 in the Supporting Information) for samples A@PEG and B@PEG (whose SAR/*f* curves are dependent on the frequency, Figures S5). In this way, the SAR(*H*) curves presented in Figure 3e indicate that sample C@PEG can produce a heat power as high as 952 W/g (at 44 mT and 142 kHz) within safety limits. This value is remarkably larger than the attainable maximum SAR_limit_ of samples A@PEG and B@PEG and, to the best of our knowledge, it is one of the highest heating performance reported so far. It should also be stressed that the calculated optimal (*Hf*)_limit_ conditions for sample C@PEG (44 mT × 142 kHz) are fully compatible with the alternating magnetic field (AMF) set-ups that are currently being used in the clinical trials for the treatment of brain, prostate, and pancreatic tumors.^33^

### 2.2 Functionalization of the Best Magnetic Core with Fluorescent Copolymers

Since the aim of the present work is to develop a nanoplatform that presents both remarkable heating power and yielding fluorescence, in this section we will assess the functionalization of the best NPs chosen in the previous section (sample C) with a fluorescent copolymer.

**Figure 4:**
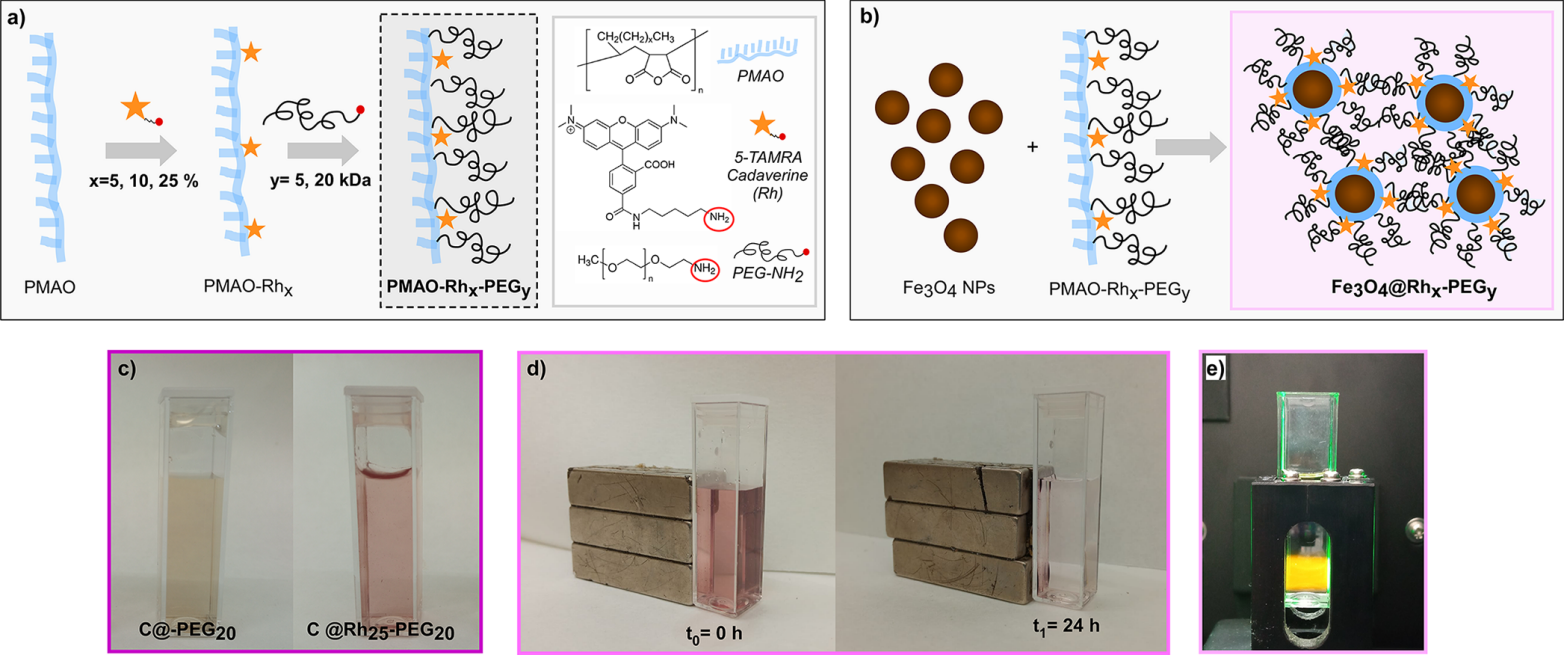
Scheme of (a) the synthesis of fluorescent-copolymers and (b) the coating procedure. Photographs showing the magneto-optic behavior of a typical C@Rh_*x*_-PEG_*y*_ sample: (c) C sample NPs with a PMAO-PEG coating (brownish colloid at 0.03 mg_Fe3O4_/mL) and with PMAO-Rh-PEG coating (pink colloid at 0.03 mg_Fe3O4_/mL), (d) a magnet close to sample C@Rh_25_-PEG_20_ initially (*t*_0_) and 24 h later (*t*_1_), and (e) fluorescence emission of sample C@Rh_25_-PEG_20_ inside the fluorimeter after excitation at 521 nm.

The PMAO-PEG copolymer used to make the samples stable in aqueous media can become fluorescent by grafting an amine modified rhodamine 3B (5-TAMRA cadaverine) into the PMAO backbone (see Materials and Methods). Several copolymers have been prepared binding variable amounts of 5-TAMRA cadaverine (Rh) to the PMAO backbone (by reacting 5, 10, and 25% of PMAO monomers) and introducing PEG molecules of two different molecular weights (5 and 20 kDa) (see Figure 4a). It has been estimated that the yield of the PMAO-Rh conjugation is around 60%, which gives rise to nanoplatforms with 300–1500 Rh molecules per NP, depending on the PMAO-Rh-PEG copolymer used in the coating (Figures S6 and S7 and Table S2).

These PMAO-Rh-PEG copolymers have been used to coat the NPs of sample C individually, as depicted in Figure 4b, following an optimized coating method.^15^ Since the NPs are individually or quasi-individually coated, the aqueous dispersions present a great colloidal stability and minimal aggregation degree (see Table S3). Additionally, the long-term stability of these NPs in PBS and cell media (DMEM) have also been demonstrated (Figure S8 and Table S4).

Samples have been named according to the amount of Rh added (*x*) and to the molecular weight of the PEG (*y*) as follows: C@Rh_*x*_-PEG_*y*_, where C represents the optimized magnetite core (sample C) and Rh_*x*_-PEG_*y*_ the polymer coating (where *x* = 5, 10, or 25% and *y* = 5 or 20 kDa). Figure 4c–e visually displays the magnetic and optical behavior that C@Rh_*x*_-PEG_*y*_ systems present. As it can be seen, there is an evident colloid color change (from brownish to pink) when Rh is incorporated to the system (Figure 4c). Moreover, the NPs are attracted toward the magnet leaving the supernatant colorless (Figure 4d), which indicates that the Rh is properly integrated within the nanoplatform and it does not leak free dye to the supernatant. In fact, to ensure that there is not free dye in the samples a thorough cleaning process has been performed using filters with appropriate pore size (see Materials and Methods). Finally, Figure 4e shows the fluorescence emission of sample C@Rh_25_-PEG_20_ (after excitation at 521 nm wavelength) in the fluorimeter. In the following, the optical properties of the whole set of C@Rh_*x*_-PEG_*y*_ samples will be analyzed in detail so as to select the surface coating that provides the best features.

### 2.3 Optical Properties of Fe_3_O_4_@Rh-PEG Systems

**Figure 5:**
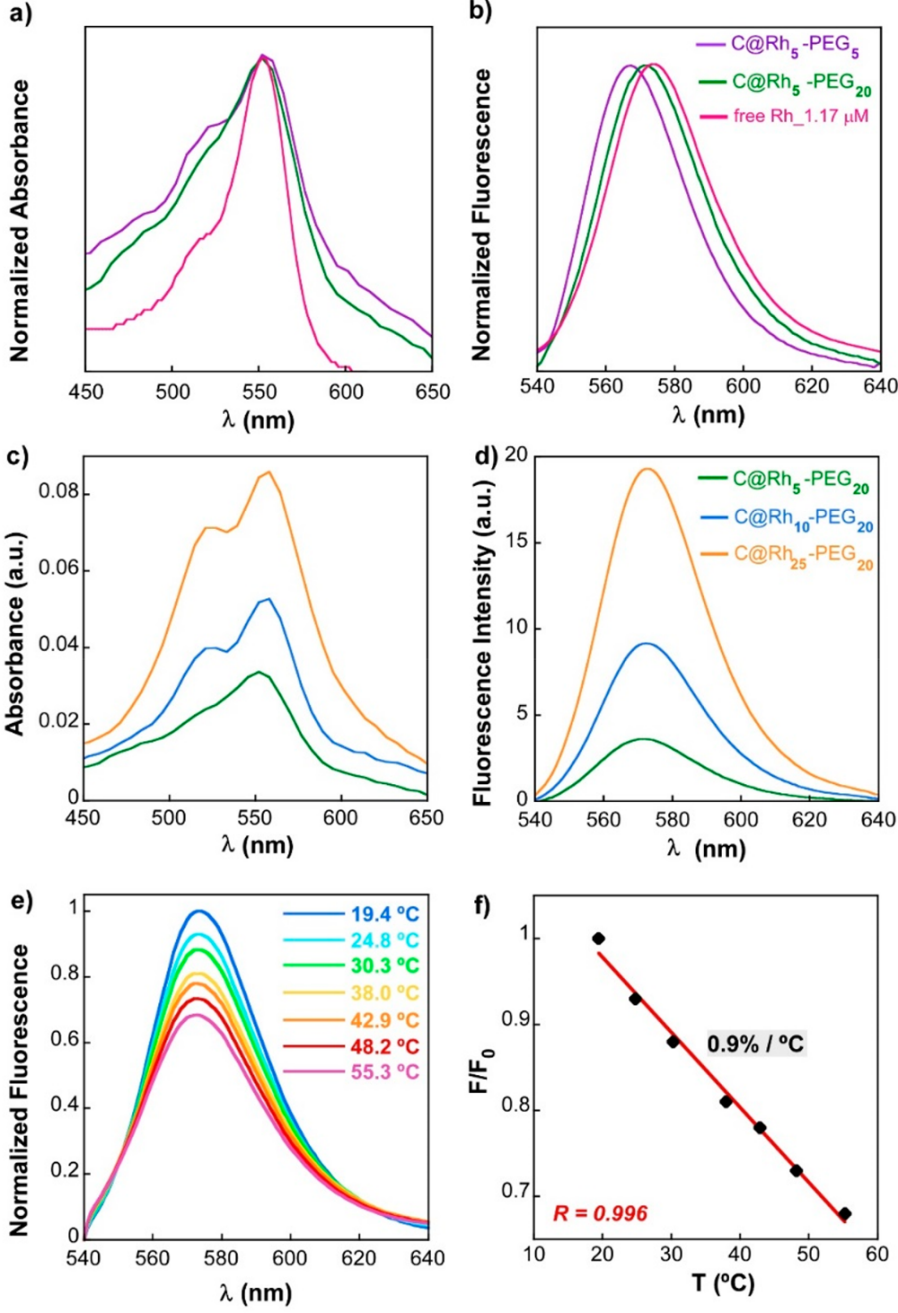
(a) Height-normalized absorption and (b) emission of 5-TAMRA cadaverine in aqueous solution (pink), and anchored within C@Rh_*x*_-PEG_*y*_ nanosystems: C@Rh_5_-PEG_5_ (purple) and C@Rh_5_-PEG_20_ (green). (c) Absorption and (d) fluorescence spectra of C@Rh_5_-PEG_20_ (green), C@Rh_10_-PEG_20_ (blue), and C@Rh_25_-PEG_20_ (orange). (e) Evolution of fluorescence spectra of sample C@Rh_10_-PEG_20_ (at 0.03 mg_Fe3O4_/mL) in aqueous solution at different temperatures. (f) Lineal thermal dependence of *F*/*F*_0_ for sample C@Rh_10_-PEG_20_. *R* refers to the Pearson correlation coefficient.

It is well-known that the insertion of dye molecules within compatible polymers can offer several advantages such as better optical homogeneity and protection from the disturbances of the external environment.^34^ However, having a structure where a dye–polymer adduct surrounds a magnetite core makes the fluorescence emission highly challenging. The goal of this section is to analyze the absorption and emission spectra of different C@Rh_*x*_-PEG_*y*_ samples in order to gain insight into the physicochemical parameters affecting the optical properties of these systems. Figure 5a, b shows the absorption and emission bands of free 5-TAMRA cadaverine (Rh) in aqueous solution, which are centered at 553 and 573 nm, respectively. From the recorded fluorescence intensity (*I*_fl_, analyzed as the area under the emission band) and the absorbance at the excitation wavelength (*A*_exc_), the fluorescence quantum yield (φ_fl_) of 5-TAMRA cadaverine was estimated to be 0.36 using a rhodamine 3B in aqueous solution as reference (see Figure S9 and Table S5). A priori requirement for an efficient fluorescent thermometer is a good enough fluorescence efficiency with a high dependence of the emission intensity on temperature changes.^23^ In this context, dyes with moderate φ_fl_ values, characterized by nonradiative deactivation rate constant (*k*_nr_) of around twice that of the fluorescence deactivation (*k*_fl_), are usually good fluorescent T-sensors. In these cases, the estimated decrease of the fluorescence emission intensities as the temperature increases is expected to be noticeable, by promoting molecular motions, increasing the probability of collisions and, therefore, favoring the nonradiative pathways.^35^ Thus, 5-TAMRA cadaverine should also be a proper candidate for a fluorescent T-sensor of local heating. Indeed, an interesting decrease of the fluorescence intensity per °C has been recorded for free 5-TAMRA cadaverine in aqueous solution, which has resulted in a high sensitivity (a reduction of the fluorescence intensity of 1.3% °C^–1^) and a wide linear working range (Figure S10). Additionally, the corresponding Arrhenius plot (see Figure S10 and Table S6) has provided an activation energy of 20.8 kJ/mol for the nonradiative deactivation process in agreement with other published values for rhodamine dyes.^36,37^ This suggests that the optical properties of 5-TAMRA cadaverine are equal to those of Rh 3B dyes, which means that the hydrocarbon spacer of 5 C atoms practically does not modify the photophysics of the fluorophore.

The optical characterization of Rh within the different C@Rh_*x*_-PEG_*y*_ samples is not a trivial task. Aqueous colloidal solutions of C@PEG nanoparticles without dye show a turbid-blackish appearance above a certain concentration of Fe_3_O_4_ NPs (*c* > 0.05 mg_Fe3O4_/mL), a fact that induces intense light scattering effects and makes the registration of the absorption spectra difficult even after applying the correction factor (i.e., subtraction of the recorded signal of C@PEG suspension without dye in the same conditions, see Figure S11 for more details). On the contrary, the effects of the light scattering of the C@Rh_*x*_-PEG_*y*_ nanosystems are not that relevant in the registration of the fluorescence band, since the incident light of the excitation beam (set at 521 nm in the present study) is not included in the wavelength emission range (525–650 nm). However, light scattering effects can reduce the penetration of the excitation beam through the sample and decrease the emitted light before arriving to the detector, inducing a decrease in the registered fluorescence intensity and leading to an underestimation of the quantum yield. Note that in the set of C@Rh_*x*_-PEG_*y*_ samples the fluorescence quantum yield (φ_fl_) parameter will not be analyzed, but rather the fluorescence efficiency (Ef_fl_), defined as the ratio between the fluorescence intensity, *I*_fl_, over the absorbance at the excitation wavelength, *A*_exc_ (Ef_fl_ = *I*_fl_/*A*_exc,_). As will be further discussed below, the fluorescence efficiency of Rh within any of the C@Rh_***x***_-PEG_*y*_ samples is 1 order of magnitude lower than that of free Rh in aqueous solution (Table 3) due to the drastic reduction in the recorded fluorescence intensity by the light scattering effect of both the Fe_3_O_4_ nanoparticles and the PMAO-PEG copolymer. The possible quenching in the fluorescence of Rh-dye by the presence of Fe^3+^ in the C@Rh_*x*_-PEG_*y*_ nanoparticle core should not be discarded either. In any case, for the standard recording conditions used in the present work, the observed emission of Rh-dye within C@Rh_***x***_-PEG_*y*_ systems can be considered good enough for biotracking (see Figure 4e). In fact, the fluorescence imaging capabilities of some of the C@Rh_*x*_-PEG_*y*_ formulations have been preliminary tested. The detected fluorescence images have indicated that the nanosystems display suitable fluorescence brightness to be clearly visualized in an optical wide-field fluorescence microscope at different Rh contents and NP concentrations (see Figure S12).

**Table 3:** Summary of the Spectral Properties of free Rh and the Studied C@Rh_*x*_-PEG_*y*_ Samples at 0.03, 0.06, and 0.12 mg/mL Concentrations of Fe_3_O_4_ NPs and 5, 10, and 25% of Rh^a^

	concentration		absorption spectra			fluorescence spectra			
sample	NPs (mg/mL)	Rh/PMAO (%)	λ_abs_ (nm)	*A*_abs_	*A*_exc_	λ_fl_ (nm)	*I*_fl_	*R*	Ef_fl_
Rh^b^			553	0.066	0.029	574	26440	2.28	911 700
C@Rh_5_PEG_5_	0.03	5	552	0.036	0.028	567	630	1.26	22 500
C@Rh_5_PEG_20_	0.03	5	552	0.034	0.024	572	681	1.40	28 400
C@Rh_5_PEG_20_^c^	0.06	5	552	0.033	0.026	572	699	1.26	26 900
C@Rh_5_PEG_20_^c^	0.12	5	552	0.055	0.045	572	1705	1.22	37 900
C@Rh_10_PEG_20_	0.03	10	552	0.050	0.038	573	1763	1.32	46 400
C@Rh_25_PEG_20_	0.03	25	556	0.086	0.071	572	3668	1.20	51 700
C@Rh_10_PEG_20_^d^PBS	0.03	10	552	0.055	0.032	573	1888	1.72	59 000
C@Rh_10_PEG_20_^d^MDEM	0.03	10	552	0.051	0.035	573	1733	1.46	49 500

a Absorption (λ_abs_, *A*_abs_, and *A*_exc_) and emission features (λ_fl_, *I*_fl_) and the parameters calculated from those spectral properties: the relationship between the absorption maxima and vibronic shoulder (*R*) and the fluorescence efficiency (E_fl_).

b The concentration of free Rh solution was 1.17 μM, which corresponds to the amount of Rh in a nanoplatform with 0.03 mg/mL NPs and 10% Rh/PMAO.

c The spectra of C@Rh_5_PEG_20_ at 0.06 and 0.12 mg_Fe3O4_/mL can be found in Figure S13.

d C@Rh_10_-PEG_20_ within physiological media (PBS) and cell media (MDEM), the corresponding spectra are displayed in Figure S14.

In order to find the C@Rh_*x*_-PEG_*y*_ system with the most suitable optical properties, the absorption and emission spectra have been analyzed in a set of samples that present different features such as the molecular weight of the PEG polymer, the concentration of Fe_3_O_4_ NPs and the relative Rh-dye/PMAO_monomer_ ratio. First of all, the effect of the PEGs tails, 5 kDa and 20 kDa, on the spectroscopic properties has been studied (keeping constant the Rh loading at 5% and the NP concentration at 0.03 mg_Fe3O4_/mL). In general terms, both C@Rh_5_-PEG_5_ and C@Rh_5_-PEG_20_ display broader absorption spectra together with a more prominent vibronic shoulder in comparison with free Rh solution (Figure 5a). These changes are similar to those typically observed in liquid solution as the dye concentration increases and are associated with the dye molecular aggregation. In fact, it is well established that the adsorption of rhodamine dyes in solid surfaces and/or in microheterogeneous systems favors the dye aggregation.^38,39^ The dye aggregation can be analyzed by means of the *R* parameter, defined as the absorbance at the maximum over that at the vibronic shoulder (Table 3). The *R* value obtained for the C@Rh_5_-PEG_20_ sample is higher than that of C@Rh_5_-PEG_5_, suggesting a lower tendency to form Rh-aggregates when a PEG of 20 kDa is used. This is an expected result, since a larger PEG chain could dispose Rh molecules to a larger distance, reducing the interaction between them and consequently the dye aggregation. Reasonably, the PEG of 5 kDa gives rise to less effective steric repulsion among magnetic cores, prompting larger NP and dye agglomeration as can be deduced from the hydrodynamic diameters determined by DLS (see Table S3).^40^ Generally, dye aggregation reduces the fluorescence efficiency, as reflected in the higher Ef_fl_ value of C@Rh_5_-PEG_20_ with respect to C@Rh_5_-PEG_5_, Table 3. Thus, in order to avoid undesirable photophysical processes and to enhance fluorescence efficiencies, we chose the PEG of 20 kDa to continue the study. In the next stage, the effect of the NP concentration was analyzed in C@Rh_5_-PEG_20__*z* samples, where *z* is the concentration of magnetite NPs in the colloid: 0.03, 0.06, and 0.12 mg_Fe3O4_/mL. The sample with the highest NP concentration (0.12 mg_Fe3O4_/mL) is very dark, significantly increasing the effect of the light scattering and making an adequate characterization of the corresponding optical properties with the equipment used virtually impossible.

Additionally, absorption spectra of the more concentrate samples (0.06 and 0.12 mg_Fe3O4_/mL, Figure S13) suggest important dye aggregation as it is reflected in the reduction of the R value (see Table 3). However, the fluorescence efficiency does not show the expected decrease with the increasing NP concentration; in fact, it is maintained nearly constant for 0.03 and 0.06 mg_Fe3O4_/mL while an increase is detected for 0.12 mg_Fe3O4_/mL. Note that the experimental errors in the C@Rh_*5*_-PEG_*20*__0.12 sample are far from negligible due to the mentioned high scattering. Considering these results, the most reasonable option is to perform the spectroscopic study of C@Rh_*x*_-PEG_*20*_ samples in the most diluted conditions, that is, 0.03 mg_Fe3O4_/mL.

Thus, to conclude with the refinement process of the magneto-optical nanoplatform, we have analyzed the relative Rh/PMAO_monomer_ ratio (or percentage) in the set of C@Rh_*x*_-PEG_20__0.03 samples. Figure 5c, d displays the absorption and emission spectra of C@Rh_***x***_-PEG_20_ at *x* = 5, 10, and 25%, respectively. Although at first glance the absorption and emission intensity appear to change consistently with the increase in Rh percentage, a closer inspection of the *A* and *I*_fl_ parameters in Table 3, show a nonlinear increase, consistent with the dye aggregation process. Indeed, the *R* value decreases by increasing Rh-content in the copolymer (Table 3), suggesting a higher dye aggregation tendency as *x* value increases. However, this is not reflected in a reduction of the fluorescence efficiency, probably because at higher relative dye concentration, the scattering and/or quenching effects become less relevant enhancing the fluorescence efficiency. In any case, if both *R* and *E*_fl_ values of C@Rh_*x*_-PEG_20__0.03 samples are taken into account, it can be confidently concluded that the sample with *x* = 10% fulfills the best compromise to achieve a cost-efficient magneto-optical nanoplatform that keeps a good fluorescent efficiency and maintains the aggregation degree at the lowest possible values (see the hydrodynamic diameters listed in the Table S3). Note that physical agglomeration among the magnetic NPs is completely critical for the magnetothermal capacity, as it has been profusely shown that the aggregation of MNPs leads to a drastic decrease in their heating power.^41^

On the other hand, for biomedical applications, it is of utmost importance to confirm that the optical properties of the refined nanoplatform remain uninfluenced when they are dispersed within physiological media. Thus, the absorption and emission spectra of C@Rh_10_-PEG_20_ in phosphate buffered saline (PBS) and in cell media (MDEM) were registered (Figure S14) and the corresponding relevant spectroscopic parameters were included in Table 3. The photophysical properties of Rh within C@Rh_10_-PEG_20_ show an optical improvement when recorded in PBS or MDEM (see Table 3). Specifically, the higher *R* value suggests a lower tendency of the dye to aggregate in PBS and MDEM, supported by the appearance of an isosbestic point at 534 nm, indicative of the coexistence of two different species, monomers and dimers. This is likely because the inclosing saline solutions in PBS and MDEM neutralize the positive charges (from Rh) and negative charges (from PMAO), canceling the intracopolymer electrostatic interaction that could cause locally compressed zones. Consequently, a more distended polymeric structure with less dye aggregation would be favored, which would in turn reduce the aggregation among magnetic NPs (Tables S3 and S4 ). It is worth mentioning the good colloidal stability of sample C@Rh_10_-PEG_20_ over time; after being stocked in the fridge for over a year, its colloidal properties do not change significantly in either PBS or MDEM (Table S4_,_Figure S8).

In order to round off the fluorescence study and determine the potential of C@Rh_10_-PEG_20_ sample as a temperature sensor, we recorded fluorescence measurements as a function of temperature in the range 20–55 °C, which covers the therapeutic temperature range of magnetic hyperthermia. Figure 5e shows the evolution of the fluorescence spectrum of C@Rh_10_-PEG_20_ with the temperature and Figure 5f suggests a linear relationship between the decrease in the fluorescence intensity maxima (*F*) and the temperature rise. The calculated sensitivity from 20 to 55 °C is 0.9% °C^–1^ (Figure 5f), which is somewhat lower than the 1.3% °C^–1^ obtained for free Rh in aqueous solution (Figure S10) but it still is a very good result if it is compared with the most promising organic fluorescent thermometers.^23,42^ In this context, considering the great complexity of this fluorescent nanoplatform composed of a Fe_3_O_4_ ferromagnetic core and a high molecular weight copolymer (>1.7 × 10^6^ Da); undoubtedly, a sensitivity of 0.9% °C^–1^ can be taken as an outstanding result. At this point, it should be stressed that counting on a local thermometer with high sensitivity, fast response and high spatial resolution is particularly advantageous in the case of a magnetothermal nanoplatform. Because local fluorescence thermometry could have the potential to self-monitor and control the temperature increase caused by the magnetic nanoparticles, which is a crucial aspect if ablation temperatures (>45 °C) need to be avoided during the hyperthermia therapy.

### 2.4 Experimental Validation of the Biomedical Potential of C@Rh_10_-PEG_20_

At this point, it is crucial to study the biocompatibility of the chosen sample and analyze if the incorporation of Rh affects the magnetothermal performance in different media.

#### 2.4.1 Viability of Sample C@Rh_10_-PEG_20_ on Cells

**Figure 6:**
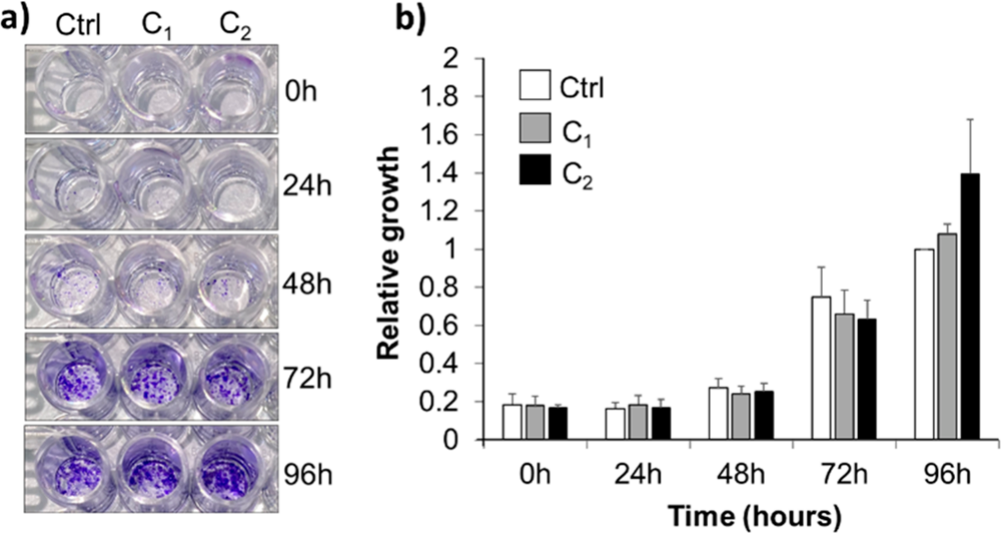
Cytotoxicity assay of cells incubated with C@Rh_10_-PEG_20_ NPs at different time points (0–96 h) using 0.1 ng_NP_/cell (*C*_1_) and 1 ng_NP_/cell (*C*_2_). (a) Crystal violet staining of one representative experiment and (b) plotted relative growth values. Values are represented as the mean and standard error of the mean of three independent experiments.

The cytotoxicity of sample C@Rh_10_-PEG_20_ at different time points ranging from 0 to 96 h was analyzed by a proliferation assay (see Figure 6a). Figure 6b shows that human colorectal cancer cells (HCT116) incubated with C@Rh_10_-PEG_20_ NPs grow at the same rate as control cells without NPs (white bar), with no significant differences between the two NP concentrations (*C*_1_ and *C*_2_, gray and black bars, respectively). Therefore, it can be concluded that this Fe_3_O_4_-rhodamine formulation is not toxic for the cells at concentrations ≤1 ng/cell.

#### 2.4.2 Verification of the Magnetothermal Efficiency of C@Rh_10_-PEG_20_

**Figure 7:**
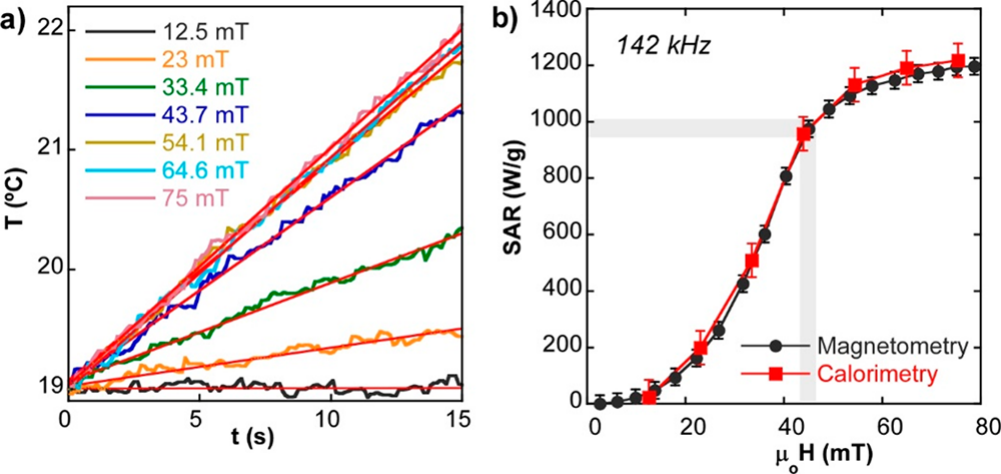
(a) Temperature increase over time at 142 kHz for different magnetic field amplitudes (12.5–75 mT) for C@Rh_10_-PEG_20_ sample in PBS. (b) SAR vs field curves obtained by AC magnetometry and by calorimetry at 142 kHz for C@Rh_10_-PEG_20_ sample in PBS. Error bars of calorimetric values have been estimated from the standard error of the linear least-square fit of the experimental (temperature, time) points. Error bars of magnetometric values have been estimated by repeatedly recording a single hysteresis loop obtained in the same excitation conditions.

The PMAO-Rh-PEG copolymer presents structural and charge distribution differences in comparison with the PMAO-PEG coating (used in the preliminary hyperthermia analysis of this work), which has an impact in the colloidal properties of the nanoparticles as it has been shown in Table S3. Therefore, a final validation of the selected magneto-luminescent platform has been carried out by measuring the hyperthermia performance of the C@Rh_10_-PEG_20_ sample by AC magnetometry and calorimetry techniques in physiological media (PBS) and using the excitation frequency of 142 kHz that maximizes the SAR of sample C, according to arguments presented in Figure 3. The calorimetry SAR values for different magnetic field amplitudes (12–75 mT) have been obtained from eq 1, by calculating the slope of the linear relation between temperature and time during the first 10 s after switching on the electromagnetic field. As shown in the Figure 7a, linearity is excellent up to at least 15 s.
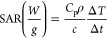
1Here, *C*_p_ is the specific heat capacity of water, ρ is the density of water, and *c* is the magnetite NP concentration. As it is shown in Figure 7b there is a very good agreement between both techniques, which reasserts the exceptional heating capacity of this sample. Moreover, the SAR obtained in sample C@Rh_10_-PEG_20_ under safe clinical conditions (≈ 1000 W/g, see gray markers in Figure 7b) is completely consistent with the previous study of sample C@PEG displayed in Figure 3e. Therefore, it can be concluded that the heating power of the C sample NPs is not impaired when using the fluorescent PMAO-Rh-PEG copolymer coating.

Additionally, it is noteworthy that the excellent magnetothermal performance of C@Rh_10_-PEG_20_ is quite insensitive to the dispersion media (it stays quite constant in cell media and in a very viscous media such as agar, Figure S15), which is essential to achieve a reliable and successful biomedical application in high-viscosity cellular environments.

## 3 Conclusions

A multifunctional platform based on Fe_3_O_4_ NPs and rhodamine 3B has been designed without disrupting the suitability that each module has separately. The size (∼25 nm) and shape (octahedral-like) of the magnetite NPs have been finely tuned in order to reach an optimal heating capacity under safe clinical conditions. It has been found that an irregular truncation in octahedral NPs confers an ideal anisotropy to the systems that leads to one of the highest magnetothermal efficiency reported so far for magnetite NPs.

To achieve a promising magneto-fluorescent nanocarrier, several copolymers formed by PMAO, PEG, and Rh have been prepared to coat the magnetite cores. The optical study has revealed that copolymers synthesized with PEG of 20 kDa and a 10% rhodamine fulfill the best compromise to achieve Fe_3_O_4_@Rh nanoplatforms with good fluorescent efficiency and minimal aggregation in different media (PBS and cell media). In addition, it has been confirmed that the fluorescence emission of the nanoplatform changes linearly with the temperature with high detection sensitivity.

To sum up, we optimized a highly biocompatible magneto-fluorescent nanosystem (based on molecular dyes and black ferromagnetic NPs) with the potential for dual-imaging, the ability to produce large heating, and the capacity for fluorescence thermometry. The archetypal theranostic platform presented herein provides an effective tool to improve the accuracy of future diagnosis and treatment of diseases.

## 4 Materials and Methods

### 4.1 Materials

Sodium oleate from TCI America (97%), ethanol from Panreac S.A, poly(ethylene glycol)-amine (PEG-NH_2_) from Laysan Bio (MW = 5000 and 20 000), and phosphate-buffered saline (PBS) from Gibco. All other solvents and reagents were purchased from Sigma-Aldrich and used as received without purification: iron(III) chloride hexahydrate (99%), oleic acid (90%), 1-octadecene (ODE) (90%), dibenzyl ether (DBE) (98%), chloroform (>99%), tetrahydrofuran (>99.9%), hexane (99%), and poly(maleic anhydride-*alt*-1-octadecene) (PMAO) (MW = 30 000–50 000 Da).

### 4.2 Synthesis of Iron Oleate

For the synthesis of iron oleate (FeOl), 40 mmol of FeCl_3_·6H_2_O and 120 mmol of sodium oleate are added to a solvent mixture (140 mL of hexane, 80 mL of ethanol, and 60 mL of D.I. H_2_O) and heated to reflux (60 °C) for 1 h under N_2_ gas. After cooling to room temperature, the aqueous phase was removed using a separatory funnel and the organic phase containing the iron oleate complex was further washed with D.I H_2_O. Finally, the organic phase with the FeOl was annealed at 110–120 °C (see Table 2) to ensure the complete removal of hexane, EtOH, and H_2_O, resulting in a black-brownish waxy solid.

### 4.3 Synthesis of Fe_3_O_4_ NPs

In a typical synthesis, the FeOl (5 mmol) was dissolved in a 2:1 mixture of organic solvents (1-octadecene, ODE, and dibenzyl ether, DBE) together with oleic acid (10–11 mmol). The mixture was heated in 2 steps under N_2_ (g) using a T controller: First, at 10 °C/min from RT to 200 °C and, second, at 3 °C/min from 200 °C to 320–325 °C. The system is kept under reflux for 60 min and then, the product is cooled to RT. The entire synthesis is carried out under mechanical stirring (at 120 rpm) and by keeping the reaction flask completely sealed. The final product is cleaned by centrifugation (20 000 rpm) using THF, EtOH, and CHCl_3_ as explained in our previous work.^13^ The stock solution is dispersed in CHCl_3_ and stored in the fridge.

### 4.4 Synthesis of PMAO-Rh-PEG Fluorescent Copolymer and Coating Procedure

Several fluorescent copolymers (PMAO-Rh-PEG) with variable content of Rh and PEG of different molecular weight were synthesized beforehand in two steps. First, 5-TAMRA cadaverine (Rh) was added to a solution of poly(maleic anhydride-*alt*-1-octadecene) (PMAO) in CHCl_3_ at different Rh/PMAO_monomer_ molar percentages: 5, 10, and 25%. PMAO and Rh solution was stirred at room temperature for 24 h. Then, poly(ethylene glycol)-amine (PEG-NH_2_) (5 or 20 kDa) was grafted into the PMAO-Rh backbone at a PEG/PMAO_monomer_ molar percentage of 75%.

The Fe_3_O_4_ NPs (sample C) were functionalized PMAO-Rh-PEG copolymers following a previously optimized protocol that avoids collective coatings.^15^ To ensure that C@Rh_*x*_-PEG_*y*_ samples did not present free dye or polymer, we first cleaned them by centrifugation (30 min at 14 000 rpm), and then by filtration using 300 kDa Amicon filters (40 min at 3500 rpm). The process was repeated, as many times as necessary, until the filtered solution was totally colorless (with no fluorescence detection).

### 4.5 Physical, Structural, and Magnetic Experimental Details

- FTIR spectra of FeOl complexes were collected on a FTIR-8400S Shimadzu spectrometer in a 4000–400 cm^–1^ range using KBr pellets.
- X-ray Diffraction (XRD) patterns of the as-synthesized dried samples were obtained using a PANalytical X’Pert PRO diffractometer equipped with copper anode (operated at 40 kV and 40 mA), diffracted beam monochromator and PIXcel detector. Scans were collected in the 10–90° 2θ range, with step size of 0.02° and scan step speed of 1.25s.
- The percentage of organic matter in as-synthesized hydrophobic NPs was determined by thermogravimetric measurements, performed in a NETZSCH STA 449 C thermogravimetric analyzer, by heating 10 mg of sample at 10 °C/min under a dry Ar atmosphere.
- TEM micrographs were obtained using a Philips CM200 with an accelerating voltage of 200 kV, and a point resolution of 0.235 nm, which provides morphology images and the corresponding structural characterization by selected-area electron diffraction.
- Quasi-static magnetization measurements as a function of magnetic field, *M*(*H*) and temperature *M*(*T*) (at 10 Oe) were carried out using a SQUID magnetometer (MPMS3, Quantum design). These measurements were performed by diluting as-synthesized NP stocks and depositing a drop on semipermeable filter paper. The saturation magnetization, *M*_s_, at RT and 5 K were obtained from dried as-synthesized samples (powder) and normalized per unit mass of inorganic matter by subtracting the weight percentage of organic matter determined by thermogravimetry.
- Absorption and fluorescence spectra were recorded in a Shmidazu UV-2600 double beam spectrophotometer and in a Shimadzu RF-5301 spectrofluorometer, respectively. Typical 1 cm optical pathway quartz cuvettes were used. The absorption and fluorescence spectra were recorded in the VIS region from 650 to 450 nm in absorption and from 540 to 640 nm in emission after excitation at 521 nm. In order to enhance the recorded fluorescence signal, relative high width slits were applied in the excitation beams (10 and 5 nm, respectively). This reduces the spectral resolution, but this is not important for Rh dye since the fluorescence spectra are characterized by a broad emission band. For temperature study, a thermostated sample holder was used in the fluorimeter, and the temperature was controlled by an external heater circulator with controllable temperature.
- The fluorescence images were obtained in an optical upright wide field microscope with epi configuration (Olympus BX51) equipped with a 120 W short arc lamp (X-Cite R Series 120Q, EXFO) as excitation source and collecting the emission with a color CCD camera (DP72). Fluorescence is excited under blue light by a D470/40 Chroma band-pass filter and the emission was clean with E515LPv2 Chroma cutoff filter, after passing through a Chroma dichroic filter 495DCLP.
- The human colorectal cancer cell line HCT116 (ATCC) was cultured in Dulbecco’s modified Eagle medium (DMEM) (Gibco) and supplemented with 10% FBS and antibiotics at 37 °C in a 5% CO_2_ atmosphere. Cells were seeded in 96-well plates at a density of 1000 cells per well and were allowed to attach to the plate before the addition of nanoparticles. After attachment, 0.1 μg of NPs (*C*_1_= 0.1 ng_NP_/cell) and 1 μg (*C*_2_ = 1 ng_NP_/cell) per well were added. Cell density was measured at 0, 24, 48, 72, and 96 h, using a crystal violet assay. In brief, cells were fixed in 4% paraformaldehyde and stained with 0.1% crystal violet. After staining, cells were washed with water and 10% acetic acid was added. Absorbance was measured at 590 nm.
- SAR (specific absorption rate) measurements have been performed by AC magnetometry in a homemade device that generates high magnetic field able to saturate the samples.^43^ This device is capable of working at a wide frequency range (100–950 kHz) with large field intensities: up to 90 mT at a low frequency limit and up to 31 mT at a high frequency limit. The dynamic hysteresis loops were measured at room temperature (25 °C) at selected frequencies of 133, 142, 304, and 634 kHz. These measurements have been carried out in PMAO-PEG and PMAO-Rh-PEG coated NPs dispersed in distilled water, in physiological media (PBS 1×) and in agar (2%). The experimental setup has been adapted to perform in situ magnetometric and calorimetric measurements. For this purpose, a sample holder includes a fiber-optic sensor immersed in the sample that allows for monitoring the temperature increase with time caused by the application of a constant radio frequency magnetic field.

## Supporting Information Available

Crystallite size, FTIR spectra, thermogravimetric measurements, ZFC-FC curves, experimental and simulated DC hysteresis loops, SAR/*f*(*H*) curves at different frequencies, determination of SAR_limit_(*H*) curves, estimation of Rh molecules attached to each NP, characterization of the colloids by DLS in different media, spectral parameters of 5-TAMRA cadaverine and Rh 3B, UV–vis absorption spectra correction, transmission/emission/overlapped images of C@Rh_*x*_-PEG_20_ systems, absorption and emission spectra at different NP concentrations and in different media, AC hysteresis loops in agar and cell media (PDF)
