# Supplementary material for: Efficient Magneto-Luminescent Nanosystems based on Rhodamine-Loaded Magnetite Nanoparticles with Optimized Heating Power and Ideal Thermosensitive Fluorescence
Source: ACS Appl Mater Interfaces. 2022 Oct 27;14(44):50033–44. doi: 10.1021/acsami.2c14016 (PMC9650688; doi:10.1021/acsami.2c14016)
Supplement: Supplementary file 1 — am2c14016_si_001.pdf [file am2c14016_si_001.pdf]

SUPPORTING INFORMATION

Efficient Magneto-Luminescent Nanosystems based on Rhodamine-loaded Magnetite Nanoparticles with Optimized Heating Power and Ideal Thermosensitive Fluorescence

Idoia Castellanos-Rubio[1]*, Ander Barón[1], Oier Luis-Lizarraga[1], Irati Rodrigo[2], Izaskun Gil de Muro[1,3], Iñaki Orue[4], Virginia Martínez-Martínez[5], Ainara Castellanos-Rubio[6,7,8,9], Fernando López-Arbeloa[5] and Maite Insausti[1,3*].

[1]Dpto. Química Orgánica e Inorgánica, Facultad de Ciencia y Tecnología, UPV/EHU, Barrio Sarriena s/n, 48940, Leioa, Spain.

[2]Dpto. Electricidad y Electrónica, Facultad de Ciencia y Tecnología, UPV/EHU, Barrio Sarriena s/n, 48940, Leioa, Spain.

[3]BC Materials, Basque Center for Materials, Applications and Nanostructures, Barrio Sarriena s/n, 48940, Leioa, Spain.

[4]SGIker, Servicios Generales de Investigación, UPV/EHU, Barrio Sarriena s/n, 48940, Leioa, Spain.

[5]Dpto. Química Física, Facultad de Ciencia y Tecnología, UPV/EHU, Barrio Sarriena s/n, 48940, Leioa, Spain.

[6]Dpto. Genética, Antropología Física y Fisiología Animal, Facultad de Medicina, UPV/EHU, Leioa, Spain.

[7]Biocruces Bizkaia Health Research Institute, Cruces Plaza, 48903, Barakaldo, Spain

[8]Biomedical Research Center in Diabetes Network and Associated Metabolic Diseases, 28029, Madrid, Spain.

[9]IKERBASQUE Basque Foundation for Science, 48013, Bilbao, Spain.

Table of contents

- **Crystallite size of Fe_3O_4 nanoparticles:**

  Table S1. Crystallite sizes of Fe_3O_4 nanoparticles.

- **FTIR spectra of FeOl precursors:**

  Figure S1: FTIR spectra of FeOl precursors.

- **Thermogravimetric measurements:**

  Figure S2. Thermogravimetry of as-synthesized NPs.

- **Magnetic characterization:**

  Figure S3. Zero field cooling and field cooling (ZFC-FC) curves.

  Figure S4. Experimental and simulated DC hysteresis loops simulations at 5 K.

  Figure S5. Experimental SAR/f versus field curves at 133, 300, and 634 kHz.

  Model S1: Determination of SAR_{limit}(H) curves

- **Estimation of Rh molecules attached to each NP:**

  Figure S6. Thermogravimetric curves of sample C coated by oleic acid and PMAO

  Figure S7. a) calibration curve of [Rh] **b)** Absorption spectra of PMAO-Rh and free-Rh.

  Table S2. Absorption area, [Rh] and Rh% in PMAO-Rh fraction and in free-Rh fraction.

- **Characterization of the colloids by Dynamic Light Scattering (DLS):**

  Table S3. Mean hydrodynamic diameter and Z Potential.

  Figure S8. Dh_N distributions of Opt_3-23@PEG in 31-05-2021 and in 05-07-2022 in PBS.

  Table S4. Dh in PBS (1x) and cell media (DMEM) for sample C@Rh_{10}-PEG_{20}

- **Optical characterization:**

  Figure S9. Absorption and emission spectra of *5-TAMRA cadaverine* and Rh 3B.

  Table S5. Spectral parameters and quantum yield of *5-TAMRA cadaverine* and Rh 3B.

  Figure S10. Normalized emission spectra (F/F_0) of *5-Tamra cadaverine* at different temperatures, lineal thermal dependence of F/F_0, and Arrhenius plot.

  Table S6. T and spectral parameters for the calculation of the E_a, associated to k_{nr}.

  Figure S11. UV-VIS spectra involved in each sample correction at 0.03 mg_{Fe3O4}/ml.

  Figure S12. A) Transmission, **B)** emission image and **C)** overlapping of both images of sample *C@Rh_5-PEG_{20}* (top) and sample *C@Rh_{10}-PEG_{20}* (bottom)

  Figure S13. Absorption and Emission spectra of C@Rh_5-PEG_{20} at ≠ concentrations.

  Figure S14. Absorption and Emission spectra of C@Rh_{10}-PEG_{20} in different media.

- **Verification of the magnetothermal efficiency of C@Rh_{10}-PEG_{20}:**

  Figure S15. AC hysteresis loops at 133 kHz of C@Rh_{10}-PEG_{20} sample in different media.

➢ Crystallite size of Fe_3O_4 nanoparticles

The crystallite sizes of samples A, B and C have been calculated by the deconvolution of the (311) diffraction peak of magnetite, using the Scherrer equation **(equation S1):**

$$D = \frac{K\lambda}{B_{struc.} cos\theta} \qquad \textbf{(S1)}$$

Where K is the shape factor (0.85-0.95), $B_{structure}$ = $B_{observed}$-$B_{instrumental}$ is the full width at half maximum, λ is the X-ray wavelength (in our case = $(K\alpha_1+K\alpha_2)/2=1.5418$Å), and θ is the peak position.

Table S1. Parameters obtained from the deconvolution of (311) of magnetite in samples A, B and C the calculated crystallite size using equation S1.

Sample	Diffraction peak	B obs. (°2θ)	B inst. (°2θ)	B estruc. (°2θ)	Peak pos. (°2θ)	Crystallite size [nm]*
A	311	0.436	0.10	0.336	35.560	25 (1)
B	311	0.427	0.10	0.327	35.590	25 (1)
C	311	0.441	0.10	0.341	35.599	25 (2)

**The deviation of the size has been obtained using K= 0.85-0.95*

➢ FTIR spectra of FeOl precursors

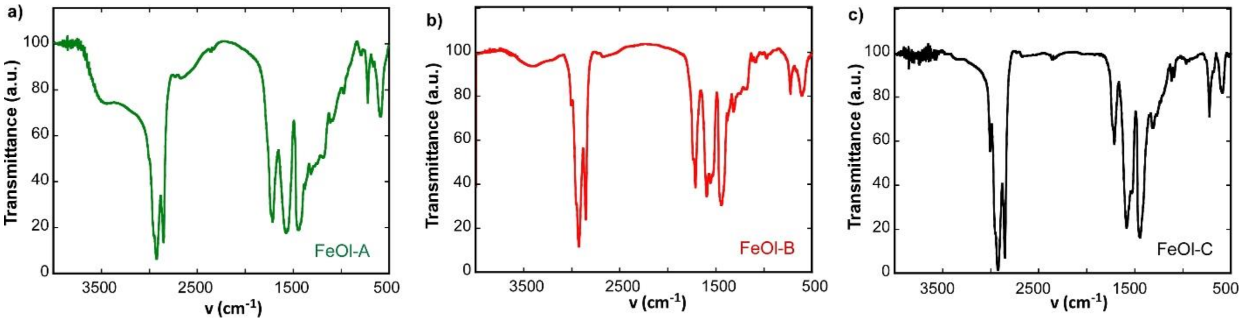

Figure S1: FTIR spectra of **a)** FeOl-A, **b)** FeOl-B and **c)** FeOl-C precursors.

➢ Thermogravimetric measurements

The as-synthesized Fe_3O_4 NPs (samples A, B and C) are coated by oleic acid. The amount of oleic acid in each sample has been determined by thermogravimetry (**Figure S2**).

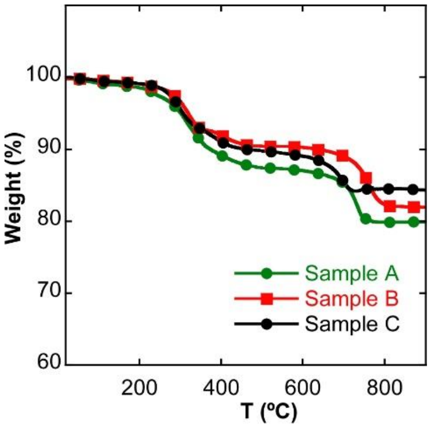

Figure S2. Thermogravimetric curves at 10 ºC/min and under Ar of as-synthesized Fe_3O_4 NPs (samples A, B and C).

The determined weight percentage of organic matter (oleic acid) in the samples is used to normalize the Ms values per unit mass of inorganic matter (Am^2/kg_{Fe3O4}).

➢ Magnetic characterization

- **Zero Field Cooling/ Field Cooling measurements**

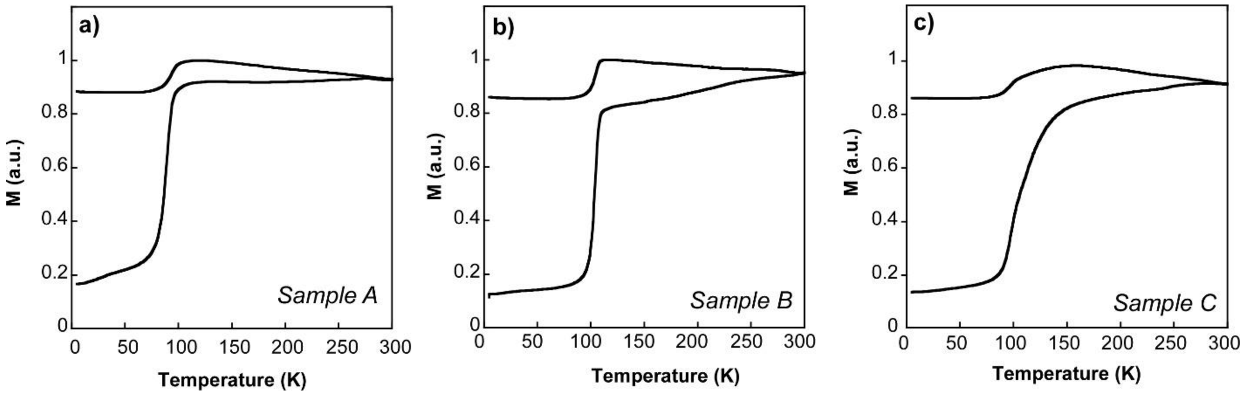

Figure S3. Zero field cooling/field cooling (ZFC-FC) curves of Fe_3O_4 NPs: **a)** A, **b)** B and **c)** C.

- **Stoner-Wolhfarth model**

DC hysteresis loops can be simulated under the approach detailed in a previous work[1] by considering a very low excitation frequency (1 Hz). The simulations that best fit the experimental loops obtained at 5 K are shown in **Figure S4**.

These simulations have given rise to different effective anisotropy constants values: K=35 kJ/m^3 for sample A, K=40 kJ/m^3 for sample B and K=47 kJ/m^3 for sample C. For simplicity, the standard deviation of the anisotropy constant distribution has been fixed to 15 kJ/m^3 in the three cases. If it is accepted that magnetite crystal lattice in the low temperature region is the same in the three samples, different values of K_{eff} mean different contributions from the shape magnetic anisotropy. As expected from TEM images, the strength of this contribution follows the relation K(A) < K(B) < K(C). As shape anisotropy changes little with temperature, this relation must be also satisfied at room temperature, what is fully consistent with SAR measurements.

(1) Castellanos-Rubio, I.; Arriortua, O.; Marcano, L.; Rodrigo, I.; Iglesias, D.; Barón, A.; Olazagoitia-Garmendia, A.; Olivi, L.; Plazaola, F.; Fdez-Gubieda, M. L.; et al. *Chem. Mater.* **2021**, *33* (9), 3139.

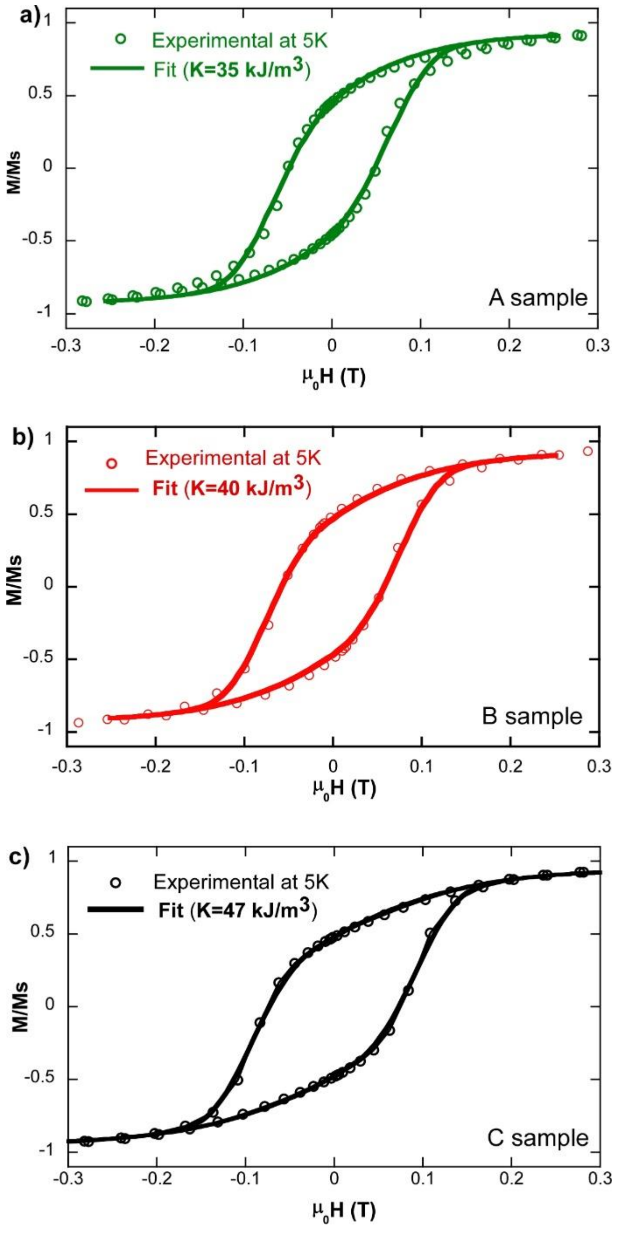

Figure S4. Experimental and simulated hysteresis loops at 5 K for Fe_3O_4 NPs: **a)** sample A, **b)** sample B and **c)** sample C.

- **AC SAR(H) curves at different frequencies**

As it can be observed in **Figure S5** the experimental SAR/f for the three recorded frequencies, which is a measure of the hysteresis area, get progressively closer when going from sample A@PEG to sample C@PEG. Indeed, SAR/f curves almost collapse for sample C@PEG meaning that in this sample the anisotropy energy (KV) becomes much larger than the thermal energy at RT. Since the three NPs (A, B, C) have very similar volume (V), these results confirm that the effective anisotropy constant of sample C at room temperature is significantly higher than those of samples A and B.

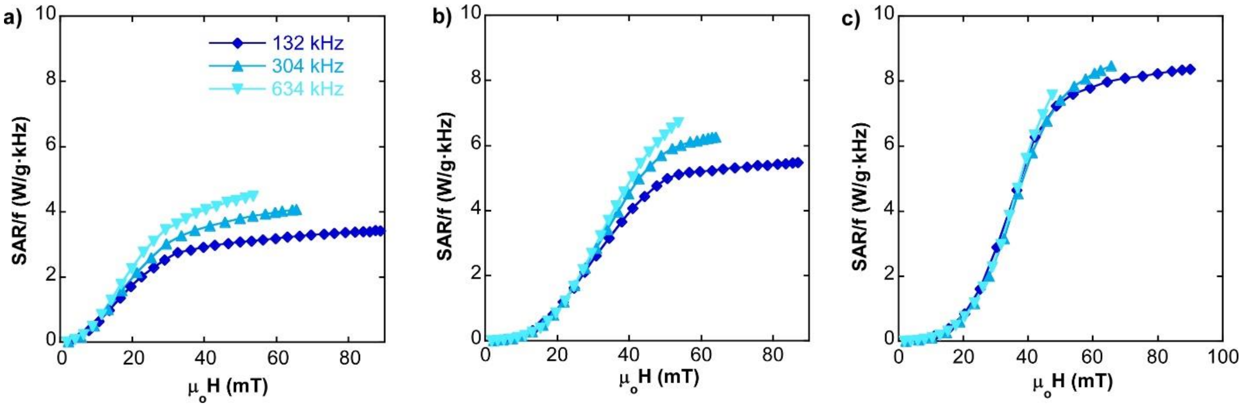

Figure S5. Experimental SAR/f versus field curves of **a)** A@PEG, **b)** B@PEG and **c)** C@PEG at different frequencies (133, 300, and 634 kHz).

- **Model S1: Determination of SAR$_{limit}$(H) curves**

In order to determine the curves of SAR$_{limit}$ versus AC magnetic field amplitude under the safe conditions imposed by Hergt criterium, we have proceeded as follows:

For samples A and B, the curves of SAR versus magnetic field amplitude (H_{ac}), corresponding to different excitation frequencies, have been fitted to a single sigmoidal-type function, dependent of some variables that are taken as linear functions of the frequency. For sample A, the selected sigmoidal-type function of H_{ac} (in units of A/m) has been the following one:

$$SAR = m1 + m_2 H_{ac} + \left[\frac{m_3}{1 + \exp(-(H_{ac} - (17/5.5)))}\right]$$

where parameters $m_1(f)$, $m_2(f)$ and $m_3(f)$ are linear function of frequency f (kHz) as:

$$m_1 = 32.74 \; - \; 0.37052 \cdot f$$

$$m_2 = -4.1567 \; + \; 0.03926 \cdot f$$

$$m_3 = -93.407 \; + \; 3.1879 \cdot f$$

In the case of sample B, the following sigmoidal function dependent of two parameters has been selected:

$$SAR = m_1(f) \cdot \left[\frac{f}{1 + 0.05 \cdot \exp(-0.14(0.00125 \cdot H_{ac} - m_2(f))}\right]$$

where parameters $m_1(f)$ and $m_2(f)$ are linear function of frequency f (kHz) as:

$$m_1(f) = 4.8 + 0.0024 \cdot f$$

$$m_2(f) = 51.8 + 0.011 \cdot f$$

The case of sample C is simpler because one may consider approximately that experimental SAR/f curves obtained at different frequencies collapse in a single one, for instance, that obtained at 134 kHz, which reaches the maximum field amplitude.

Finally,SAR_{limit} as a function of the excitation field amplitude (H_{ac}) is calculated as the f_{limit}times the SAR/f for each value of H_{ac}:

$$SAR_{limit} = f_{limit} \cdot SAR/f$$

where f_{limit} in kHz is fixed by the Hertg criterium:

$$f_{limit}(kHz) = \frac{5 \times 10^6}{H_{ac}}$$

➢ Estimation of Rh molecules attached to each NP

In order to estimate the number of Rh molecules per nanoparticle, firstly, the number of PMAO monomers on the surface of the NPs must be calculated. To do so, thermogravimetric analysis has been carried out in clean as-synthesized sample C (C@OleicAcid in **Figure S6**). The black curve indicates the amount of oleic acid on the surface of the NPs of sample C, which corresponds to ~3 oleic acid molecules/nm^2 (taking into account cuboctahedrons of Fe_3O_4 with an average dimension of 23 nm).

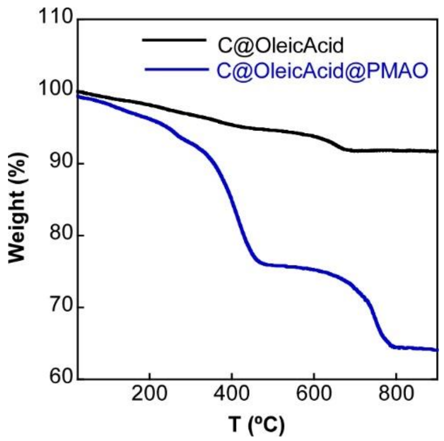

Figure S6. Thermogravimetric curves at 10 ºC/min and under Ar of as-synthesized sample C coated by oleic acid (black curve) and sample C coated by oleic acid and PMAO polymer (blue curve).

The same approach has been used to estimate the number of PMAO monomers on the surface. In this case, the contribution of oleic acid has to be subtracted from the total weight loss displayed in the blue curve (C@OleicAcid@PMAO in Figure SX). In this way, the calculated amount of PMAO monomers per nm^2 of NP surface is ~7, which means that there are around 10.000 PMAO monomers per NP.

The next step is to calculate the yield of the conjugation between 5-Tamra cadaverine (Rh) and PMAO. For this purpose, after reacting Rh with PMAO, a thorough cleaning process has been performed (using Amicon filters of 10 kDa) to separate the conjugated PMAO-Rh fraction from the free-Rh fraction.

A calibration curve has been built to determine the concentration of Rh in a linear range of 0.1-1 µg/mL, taking into account the area under the absorption signal (see **Figure S7a**).

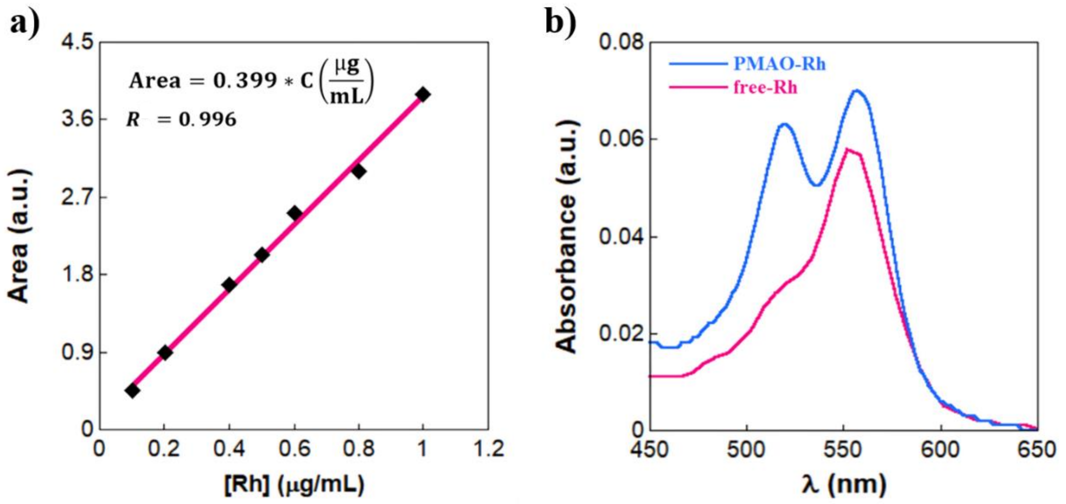

Figure S7. a) calibration curve in a [Rh] range of 0.1-1 μg/mL, **b)** Normalized absorption spectra of PMAO-Rh and free-Rh.

The absorption spectra of the PMAO-Rh and the free-Rh have been registered (see **Figure S7b**), and by using the calibration curve, the Rh concentration in the PMAO-Rh fraction and in the free-Rh fraction has been determined (see **Table S2**). In this way, it has been estimated that the Rh % that has been conjugated to the PMAO is around 60 %.

Table S2. Absorption area, [Rh] and Rh % in the PMAO-Rh fraction and in the free-Rh fraction.

Sample	Absorption Area	[Rh] ($\mu g \cdot ml^{-1}$)	Rh %
PMAO-Rh fraction	**2.65**	**0.89**	**60**
free-Rh fraction	**1.65**	**0.57**	**40**

Thus, in the PMAO-Rh_{10} copolymer where the 10 % of Rh has been initially added, experimentally the 6 % of Rh is anchored. Since it is assumed that the number of PMAO monomers surrounding the NPs surface is similar when using PMAO-Rh-PEG copolymers, it can be concluded that sample C@Rh_{10}-PEG_{20} would have around 600 Rh molecule per NP. Accordingly, C@Rh_{5}-PEG_{20} would have around 300 Rh molecule per NP and C@Rh_{25}-PEG_{20} around 1500 Rh/NP.

➢ Characterization of the colloids by Dynamic light scattering (DLS)

The measurement of Z potential (P_z) and hydrodynamic diameter (D_{hN}) of samples C@Rh_5-PEG_5, C@Rh_5-PEG_{20}, C@Rh_{10}-PEG_{20} and C@Rh_{25}-PEG_{20} have been summarized in **Table S3**. The negative Z potential values in all the samples come from the excess of COO^- groups in the PMAO-PEG-Rh copolymer.

Table S3. Polydispersity Index (PDI), Mean hydrodynamic diameter (given in Number (D_{hN})) and Z Potential for samples C@Rh_5-PEG_{20} (in D.I. H_2O), C@Rh_{10}-PEG_{20} (in D.I. H_2O and PBS) and C@Rh_{25}-PEG_{20} (in D.I. H_2O).

Sample	PDI	D_{hN} (nm)	Pz (mV)
C@Rh_5PEG_5 (in H_2O)	0.440	107 (1)	-29 (2)
C@Rh_5PEG_{20} (in H_2O)	0.328	95 (5)	-25 (1)
C@$Rh_{10}PEG_{20}$ (in H_2O)	0.250	78 (6)	-21 (1)
C@$Rh_{25}PEG_{20}$ (in H_2O)	0.331	94 (2)	-25 (1)
C@$Rh_{10}PEG_{20}$ (PBS)	0.213	64 (10)	-

Reasonably, the sample C@Rh_5PEG_5 with smaller PEG (5kDa) has a higher D_h due to less effective steric repulsion among magnetic cores. As it can be observed in **Table S3**, the PDI and the D_{hN} improve for the samples with a PEG of 20 kDa.

C@$Rh_{\mathbf{10}}PEG_{20}$ presents lower PDI and D_h in comparison to C@$Rh_{\mathbf{5}}PEG_{20}$, which could be due to a suitable amount of Rh in the copolymer that acts as a molecular hindrance prompting a more extended polymeric structure and favoring a better steric repulsion.

Otherwise, the values of PDI and D_h values of sample C@$Rh_{\mathbf{25}}PEG_{20}$ increase in relation to C@$Rh_{\mathbf{10}}PEG_{20}$. This is probably because the addition of 25 % of Rh into the PMAO backbone hampers the bonding among PMAO and PEG (which is intended to be the 75 %). And a lower amount of PEG in the copolymer entails less spacer capacity and, thus, larger aggregation.

Finally, the best colloidal properties have been obtained in C@$Rh_{\mathbf{10}}PEG_{20}$ sample in PBS. This seems to be because the phosphate saline solution neutralizes the positive charges (coming from Rh) and negative charges (coming from the PMAO) canceling the intramolecular electrostatic interaction that could cause locally compressed zones. Consequently, a more distended polymeric structure would be favored, reducing the aggregation among magnetic NPs.

- **Long-term stability of C@Rh*10*-PEG*20***

Sample C@Rh*10*-PEG*20* dispersed in PBS (1x) has been stocked in the fridge for over a year and its colloidal properties have been rechecked. The DLS analysis of C@Rh*10*-PEG*20* in 31-05-2021 and in 05-07-2022 are quite equivalent (**Table S4**), which proves the excellent long-term stability of these nano-platforms in physiological conditions (PBS). In order to better illustrate the small changes of the colloidal properties of C@Rh*10*-PEG*20* sample over time, the Dh_N distributions (in PBS) obtained in 2021 and in 2022 have been plotted together and are displayed in **Figure S8.**

Table S4. Mean hydrodynamic diameter (given in Intensity (Dh_I), Volume (Dh_V) and Number (Dh_N)) in PBS (1x) and cell media (DMEM) for sample C@Rh*10*-PEG*20*. The set of measurements have been repeated one year later to analyze the colloidal stability of the sample.

SAMPLE	Disersion media	DLS (nm) D_h (σ)			Date
		Intensiy	Volume	Number	
C@Rh*10*-PEG*20*	PBS	140 (8)	117 (6)	64 (10)	31-05-2021
C@Rh*10*-PEG*20*	PBS	135 (24)	116 (10)	98 (4)	05-07-2022
C@Rh*10*-PEG*20*	Cell media	162 (13)	140 (9)	79 (7)	05-07-2022

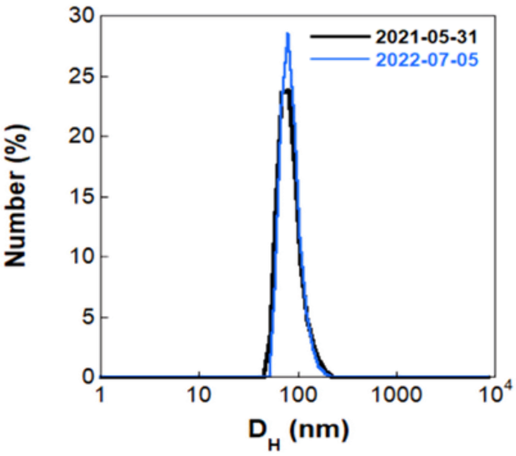

Figure S8. Dh_N distributions of Opt$_3$-23@PEG in 31-05-2021 and in 05-07-2022 in PBS.

- **Colloidal stability of C@Rh*10*-PEG*20* in cell media**

The colloidal properties of C@Rh*10*-PEG*20* sample has been further investigated in cell media DMEM (Dulbecco's Modified Eagle Medium supplemented with 10% FBS) in order to analyze the stability of the colloid in a more biological environment.

As it can be observed in **Table S4** the aggregation degree in both PBS and cell media is quite similar, concluding that C@Rh*10*-PEG*20* sample maintains its good colloidal properties when it is also within a biological environment.

➢ Optical characterization

- **Calculation of fluorescence quantum yield (ϕ_{fl}) of *5-TAMR*A cadaverine**

The fluorescence quantum yield (ϕ_{fl}) of *5-TAMRA cadaverine* has been calculated from the fluorescence intensity (I_{fl}, evaluated as the area under the emission band) and the absorbance at the excitation wavelength (A_{exc}) by means of the relative method[2], in which a standard compound of known fluorescence quantum yield is used as reference, Rhodamine 3B (Rh 3B). For low A_{exc} values (let's say < 0.2) and if experimental data of sample and reference are recorded in the same conditions (including solvent), the quantum yield is calculated by **equation S2**:

$$\phi_{fl} = \phi_{fl}^{r} * \frac{I_{fl}}{I_{fl}^{r}} * \frac{A_{exc}^{r}}{A_{exc}} \quad \textbf{(S2)}$$

where superscripts "r" corresponds to the reference, a diluted aqueous solution of Rhodamine 3B ($\phi_{fl}^{r} = 0.19$ [3]) in the present work.

The absorption and emission spectra of Rh 3B and *5-TAMRA cadaverine* can be seen in **Figure S9** and the corresponding spectral parameters and quantum yields have been listed in **Table S5.**

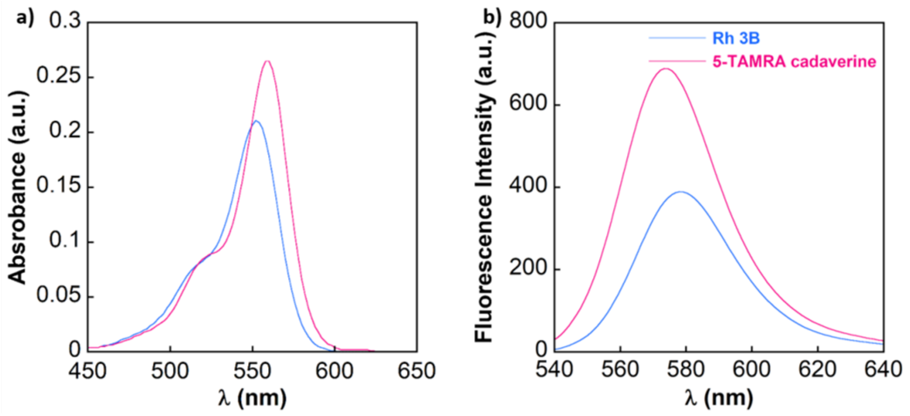

Figure S9. a) Absorption spectra and b) emission spectra of water solutions of *5-TAMRA cadaverine* at 5.84 μM and Rh 3B, diluted in situ to match vibronic shoulders. λ_{exc} = 521 nm.

Table S5. Spectral parameters and quantum yield of *5-TAMRA cadaverine* and Rh 3B in H_2O.

**Sample**	**λ_{abs} (nm)**	**A_{max}**	**A_{exc}**	**λ_{fl} (nm)**	**I_{fl}**	**ϕ_{fl}**
5-TAMRA cadaverine	553	0.265	0.083	574	26400	0.36
Rhodamine 3B	549	0.211	0.084	578	14800	0.19 [3]

(2) Hishida, I.; Bünzli, J. C. G.; Beeby, A. *Pure Appl. Chem.* **2016**, 88, 701–711.

(3) López Arbeloa, F.; López Arbeloa, T; Tapia Estévez, M.J.; López Arbeloa, I. *J Phys. Chem.* **1991**, 93, 2203-2208.

- **Thermosensitivity of free 5-Tamra cadaverine**

The fluorescence quantum yield (ϕ_{fl}) can depend on the temperature (T) through the non-radiative deactivation. Indeed, since spectroscopic transitions do not have associated any activation energy (E_a), the fluorescence deactivation rate constant (k_{fl}) is independent of the temperature, whereas the non-radiative rate constant (k_{nr}) has associated an E_a given by the corresponding Arrhenius **equation S3**:

$$k_{nr} = A_{nr}\, exp(-\frac{E_a}{R\,T}) \qquad \textbf{(S3)}$$

where A_{nr} is the preexponential factor and R, the molar gas constant.

The logarithmic version is displayed in **equation S4**:

$$\ln(k_{nr}) = \ln(A_{nr}) - \frac{E_a}{R\,T} \qquad \textbf{(S4)}$$

in which a linear relationship between ln (k_{nr}) and $1/T$ is stablished, and from its slope the corresponding E_a value can be obtained.

Since the quantum yield is defined as follows (**equation S5**):

$$\phi_{fl} = \frac{k_{fl}}{k_{fl}+k_{nr}} \qquad \textbf{(S5)}$$

Then, k_{nr} can be correlated with ϕ_{fl} by **equation S6**:

$$k_{nr} = k_{fl}\left(\frac{1}{\phi_{fl}} - 1\right) \qquad \textbf{(S6)}$$

Consequently, the E_a value can be obtained from the slope of the linear relationship between $\ln\left(\frac{1}{\phi_{fl(T)}} - 1\right)$ and $1/T$.

Figure S10a shows the experimental decrease of the fluorescence with the temperature and **Figure S10b** and **S10c** show the linear thermal dependence of F/F_0 and the corresponding Arrhenius plot, respectively. **Table S6** displays the values used for the calculation of activation energy (E_a) for the non-radiative deactivation process of *5-TAMRA cadaverine*. The lineal thermal dependen F/F_0 has provided a sensitivity of 1.3 % $°C^{-1}$ while the Arrhenius plot has provided an E_a of 20.8 kJ/mol.

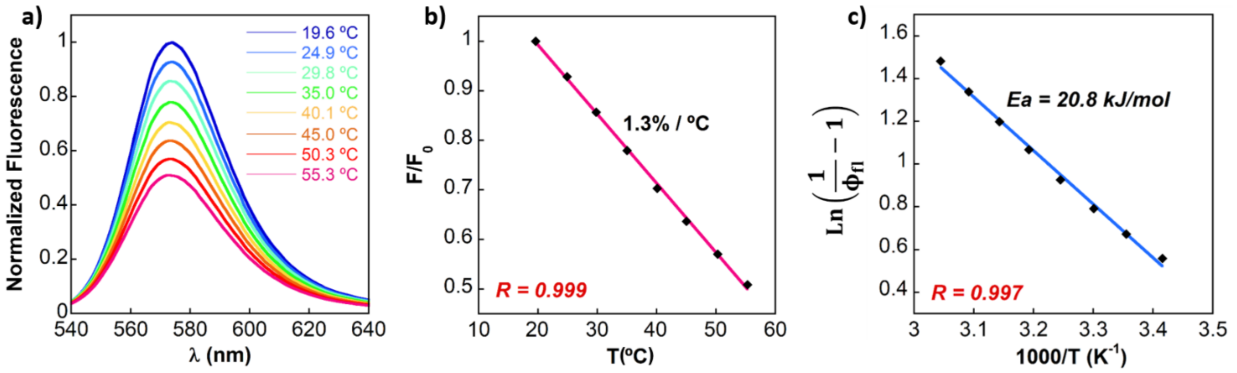

Figure S10. a) Normalized fluorescence emission spectra of *5-Tamra cadaverine* at different temperatures in aqueous solution. **b)** linear thermal dependence of F/F_0, **c)** linear relationship between $ln(k_{nr})$ and $1/T$, correlated through ϕ_{fl}. *R* refers to Pearson Correlation Coefficient.

Table S6. Temperature and spectral parameters for the calculation of the E_a, associated to k_{nr} of *5-TAMRA cadaverine* in aqueous solution.

I_{fl}	T (˚C)	T (K)	1000/T (K^{-1})	ϕ_{fl}	Ln (1/ϕ-1)
42	19.6	292.8	3.42	0.36	0.56
39	24.9	298.1	3.36	0.34	0.67
37	29.8	303.0	3.30	0.31	0.79
34	35.0	308.2	3.25	0.28	0.93
31	40.1	313.3	3.19	0.26	1.07
28	45.0	318.2	3.14	0.23	1.20
25	50.3	323.5	3.09	0.21	1.34
23	55.3	328.5	0.05	0.19	1.48

- **UV-VIS absorption spectra correction**

To extract the contribution of the scattering produced by the Fe_3O_4 nanoparticles and the PMAO and PEG polymers in C@Rh_x-PEG_y samples, the spectra of equivalent sample without Rh (C@ PEG_y) was measured at given concentration (0.03, 0.06 or 0.12 mg_{Fe3O4}/mL).

The spectra of C@ PEG_y is multiplied by a random factor (F), so it adjusts as much as possible to the spectra to be corrected (*C@Rh_x-PEG_y), specifically in the range where absorption occurs (450-650 nm). Finally, the spectra of C@PEG_{20} is subtracted from the spectra of the C@Rh_x-PEG_y samples, obtaining the corrected spectra. **Figure S11** shows some examples of the correction process.

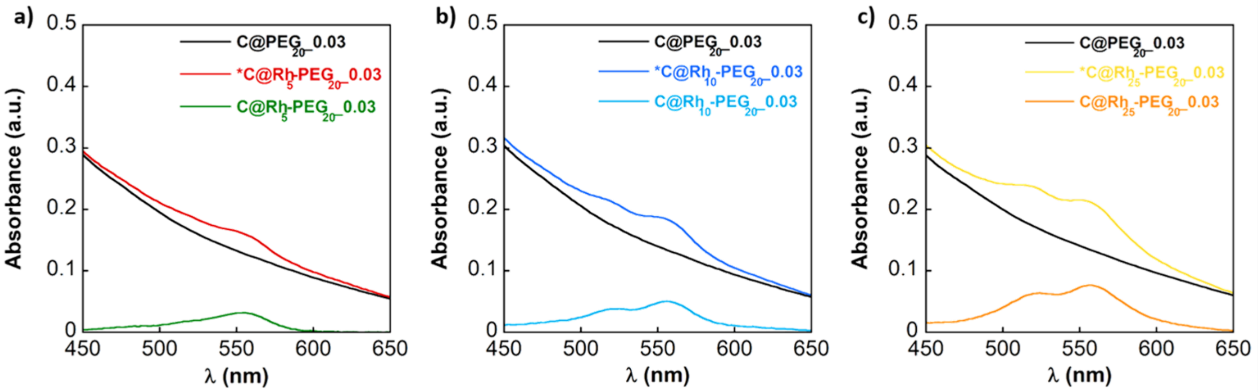

Figure S11. UV-VIS spectra involved in each sample correction at 0.03 mg_{Fe3O4}/ml a) C@Rh_5-PEG_{20}_0.03 (F=0.705). b) C@Rh_{10}-PEG_{20}_0.03 (F=0.360). c) C@Rh_{25}-PEG_{20}_0.03 (F=0.318).

- **Fluorescence imaging capability of C@Rh$_x$-PEG$_y$ systems**

Transmission and emission images of C@Rh$_x$-PEG$_{20}$ samples with different content of rhodamine (x= 5 % and 10 %) and at different Fe_3O_4 NP concentrations (c=0.03 mg$_{Fe3O4}$/ml and 0.003 mg$_{Fe3O4}$/ml) have been registered (see **Figure S12**), confirming the suitability of these systems for fluorescence imaging.

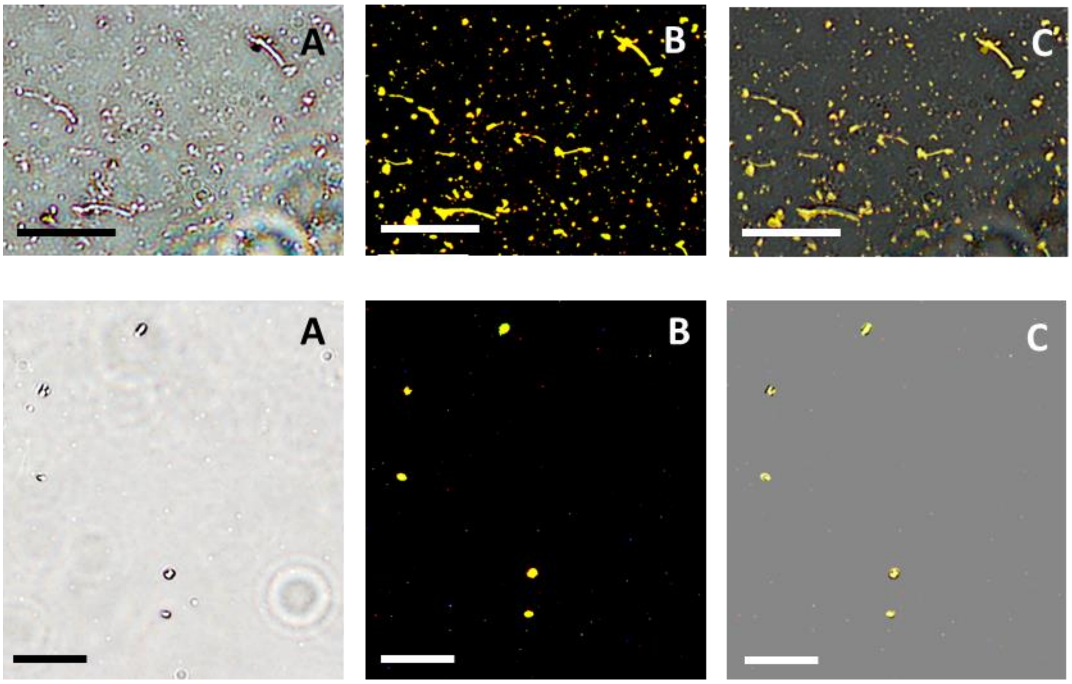

Figure S12. A) Transmission, **B)** emission image and **C)** overlapping of both images of sample *C@Rh5-PEG20 at 0.03 mg/ml* (top) and sample *C@Rh$_{10}$-PEG20 at 0.003 mg/ml* (bottom). Scale bars: 2 μm

- **Absorption and emission spectra at higher concentrations**

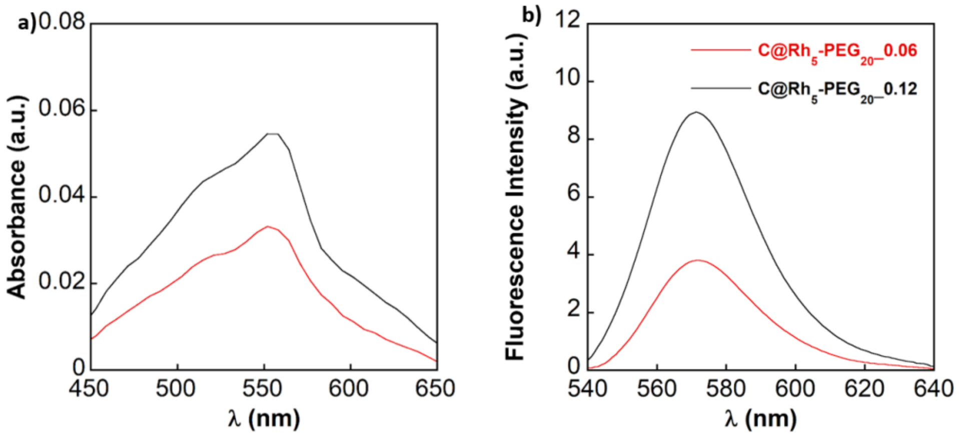

Figure S13. a) Absorption and **b)** Emission spectra of C@Rh_5-PEG_{20} at 0.06 and 0.12 mg_{Fe3O4}/ml in H_2O.

- **Absorption and emission spectra of C@Rh_{10}-PEG_{20} in H_2O, PBS and MDEM.**

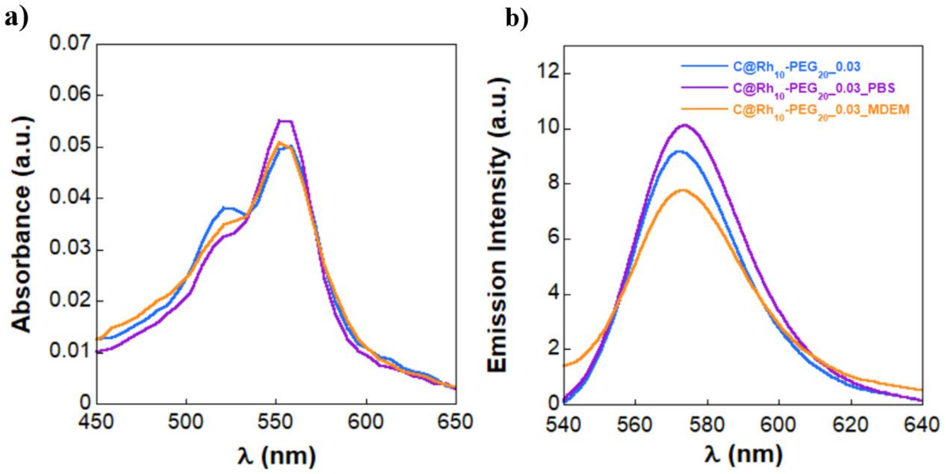

Figure S14. a) Absorption and **b)** Emission spectra of C@Rh_{10}-PEG_{20} at 0.03 mg_{Fe3O4}/ml in H_2O (blue line), PBS (purple line) and MDEM cell media (orange line).

➢ Verification of the magnetothermal efficiency of $C@Rh_{10}$-PEG_{20}

Figure S15 shows the AC loops of optimal $C@Rh_{10}$-PEG_{20} sample in PBS, Agar and cell media.

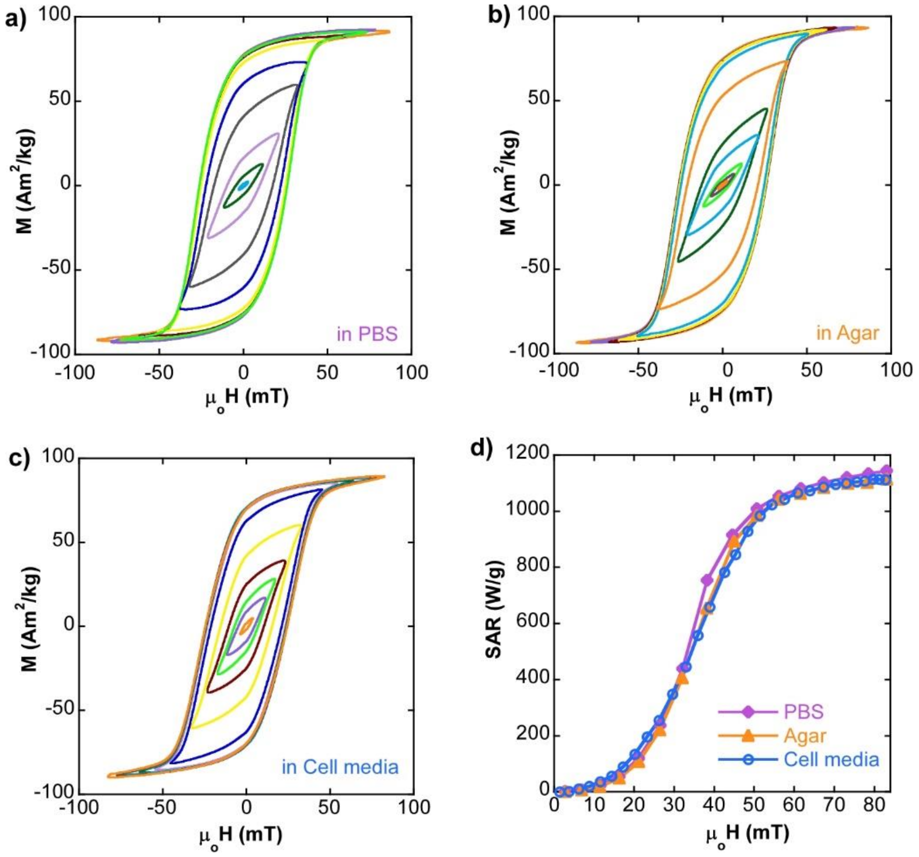

Figure S15. AC hysteresis loops at 133 kHz of $C@Rh_{10}$-PEG_{20} nanoparticles in **a)** PBS, **b)** immobilized in agar, **c)** in DMEM cell media and **d)** The corresponding SAR vs field curves.
